# Plasmin and sterile inflammation jointly drive fatal embryonic liver degeneration in endothelial chromatin remodeler mutant mice

**DOI:** 10.1242/dev.205538

**Published:** 2026-05-28

**Authors:** Meng-Ling Wu, Yelyzaveta Rudenko, Courtney T. Griffin

**Affiliations:** ^1^ Cardiovascular Biology Research Program, Oklahoma Medical Research Foundation, Oklahoma City, OK 73104, USA; ^2^ Department of Cell Biology, University of Oklahoma Health Sciences Center, Oklahoma City, OK 73104, USA

**Keywords:** Endothelial cells, BRG1, CHD4, uPAR, ICAM1, TNF

## Abstract

Embryonic livers undergo extensive vascular expansion after midgestation to support rapid growth and evolving functions. Immature embryonic vessels receive structural support from extracellular matrix (ECM), which is also essential for normal liver development and function. Meanwhile, pro-inflammatory cytokines that promote hematopoiesis and hepatic organogenesis must be tightly regulated to prevent sterile inflammation. However, endothelial contributions to ECM and cytokine production during liver development remain poorly understood. Here, we demonstrate that the chromatin remodelers CHD4 and BRG1 act antagonistically in embryonic endothelial cells to protect developing livers from lethal degeneration. Transcriptomic analysis of endothelial *Chd4* mutant livers revealed increased activity of plasmin (an ECM protease) and sterile inflammation before the onset of overt phenotypes. Within these pathways, we found that endothelial CHD4 and BRG1 antagonistically regulate transcription of the plasmin activator uPAR and of the inflammatory adhesion molecule ICAM1 in developing livers. Our genetic and pharmacological data demonstrate that elevated plasmin activity and sterile inflammation synergistically contribute to hepatic degeneration. These findings highlight endothelial transcriptional control of plasmin activity and sterile inflammation and reveal their detrimental synergy during liver development.

## Teaser

**Summary:** Embryonic mouse mutants lacking endothelial chromatin remodelers show aberrantly elevated plasmin activity and sterile inflammation that drive lethal liver degeneration, revealing endothelial programs essential for normal liver development.

## INTRODUCTION

The hepatic vascular system develops in coordination with liver growth during embryogenesis (Augustin and Koh, 2017; Lorenz et al., 2018). In mouse embryos, the formation of two key hepatic vascular structures, sinusoidal capillaries and portal veins, begins around embryonic day (E) 10 (Soares-da-Silva et al., 2020). At this stage, the developing liver vasculature is immature and vulnerable to disruptions in the extracellular matrix (ECM), a structural component that is crucial for maintaining vascular integrity (Pöschl et al., 2004; Davis and Senger, 2005; Chang et al., 2006; Delgado-Olguín et al., 2014). Meanwhile, the liver is the primary hematopoietic organ during embryogenesis, providing a site for hematopoietic stem cells to colonize and mature (Soares-da-Silva et al., 2020). Liver sinusoids must maintain their structure to effectively accommodate the translocation of hematopoietic progenitor cells and blood cells between the liver parenchyma and circulation, all while preserving their barrier function. Additionally, the ECM is essential for supporting hepatic development and homeostasis (Amenta and Harrison, 1997; Boulter et al., 2013; Goddi et al., 2021). Abnormalities in the ECM can disrupt liver cell differentiation, create an imbalance in the hematopoietic niche, and trigger cellular necrosis (Löhler et al., 1984; Gupta et al., 1998; Lorenzini et al., 2010; Tanimizu et al., 2012; Kourouklis et al., 2016; Soares-da-Silva et al., 2020; Mahony et al., 2021).

Another factor that compromises developing liver vasculature and contributes to lethal hepatic degeneration is excessive sterile inflammation. This conclusion emerged from observations that mouse embryos lacking components of the NFκB signaling pathway, such as p65 (RELA), IKKβ (IKBKB) and NEMO (IKBKG), died with liver degeneration after midgestation (Beg et al., 1995; Li et al., 1999; Rudolph et al., 2000). Notably, deletion of the inflammatory cytokine tumor necrosis factor α (TNFα) or its receptor TNFR1 (TNFRSF1A) alongside these NFκB pathway components prevented embryonic liver degeneration (Li et al., 1999; Doi et al., 1999; Rudolph et al., 2000; Rosenfeld et al., 2000). This is a developmental stage at which the liver is the main site of hematopoiesis (Soares-da-Silva et al., 2020), so sterile inflammation in these studies likely arose *in situ* within the liver rather than via infiltration of inflammatory cells from other sites. These findings contributed to the current understanding that TNFα is a multifunctional cytokine, which promotes cell proliferation, differentiation and survival during liver organogenesis, in addition to facilitating hepatic inflammation and cell death under postnatal challenge conditions (Kamiya and Gonzalez, 2004; Brenner et al., 2015; You et al., 2021; Tiegs and Horst, 2022). Specifically, the genetic studies listed above revealed that TNFα-mediated NFκB signaling aids normal liver development in the sterile embryonic environment, although TNFα signaling results in embryonic hepatic cell death when the NFκB pathway is compromised.

ATP-dependent chromatin-remodeling complexes have emerged as pivotal regulators of vascular integrity during embryonic development, operating in spatially and temporally specific manners (Ingram et al., 2013; Crosswhite et al., 2016; Menendez et al., 2017). These complexes utilize ATP hydrolysis to modulate chromatin accessibility, thereby shaping gene expression programs. Our previous work demonstrated that endothelial-specific deletion of CHD4 (chromodomain helicase DNA binding protein 4), the ATPase subunit within the NuRD (nucleosome remodeling and deacetylase) complex, leads to excessive activation of the protease plasmin, hepatic ECM degradation, sinusoidal rupture and liver degeneration, culminating in embryonic lethality by E16.5 (Crosswhite et al., 2016). Plasmin, which is generated from the circulating zymogen plasminogen via tissue- or urokinase-type plasminogen activators (tPA or uPA), is a key mediator of ECM remodeling and clot resolution (Smith and Marshall, 2010). The uPA receptor (uPAR; PLAUR) facilitates and localizes the plasmin activation process. We found that *Chd4* endothelial mutants have increased transcription of the uPAR gene (*Plaur*), which correlates with elevated hepatic plasmin activity (Crosswhite et al., 2016). This heightened plasmin activity is consistent with our discovery of diminished expression of the ECM protein laminin, a direct target of plasmin proteolytic activity and a rich component of the basement membrane surrounding sinusoidal capillaries in embryonic livers (Crosswhite et al., 2016). We therefore concluded that endothelial CHD4 transcriptionally inhibits excessive plasmin generation to protect the integrity of embryonic liver vasculature.

Different chromatin-remodeling complexes can act on shared genomic targets in cooperative or antagonistic ways (Ramirez-Carrozzi et al., 2006; Curtis and Griffin, 2012; Morris et al., 2014; Shi et al., 2023; Wu et al., 2024). Like CHD4, Brahma-related gene 1 (BRG1; SMARCA4), the ATPase in the SWI/SNF (switch/sucrose non-fermentable) complex, crucially contributes to gene transcription in developing embryonic vasculature (Griffin et al., 2011; Davis et al., 2013; Menendez et al., 2017). Notably, BRG1 co-occupies a significant number of genomic regions with CHD4, as revealed by chromatin immunoprecipitation (ChIP) sequencing (Morris et al., 2014). We previously found that simultaneous deletion of both *Brg1* and *Chd4* in endothelial cells (ECs) allows embryonic survival until birth, despite causing defects in lung development (Wu et al., 2024). However, whether transcriptional modification of plasmin activation or of other biological processes allows *Brg1/Chd4* double mutants to bypass the lethal liver phenotypes observed in *Chd4* single mutants remains unclear.

In this study, we found that the survival of *Brg1/Chd4* double mutant embryos was transcriptionally mediated by the suppression of both aberrant plasmin activation and sterile inflammation in fetal livers. We show that *Brg1* and *Chd4* act antagonistically in regulating the transcription of key components of these pathways in ECs during liver development: *Plaur* and intercellular adhesion molecule 1 (*Icam1*). While genetic deletion of *Plg* (the gene encoding plasminogen) or treatment with the anti-inflammatory drug carprofen alone was insufficient to rescue embryonic lethality in *Chd4* mutants, co-treatment with carprofen in *Plg-*deficient *Chd4* mutants successfully improved liver degeneration and embryonic survival. These findings reveal dual transcriptional roles for ECs in regulating plasmin activity and sterile inflammation in embryonic livers, thereby highlighting the detrimental and synergistic effects of these processes on hepatic development and maintenance.

## RESULTS

### Cell death and erythrocyte accumulation seen in *Chd4-ECko* mutant livers are rescued in *Brg1/Chd4-ECdko* mutants

**Fig. 1.**
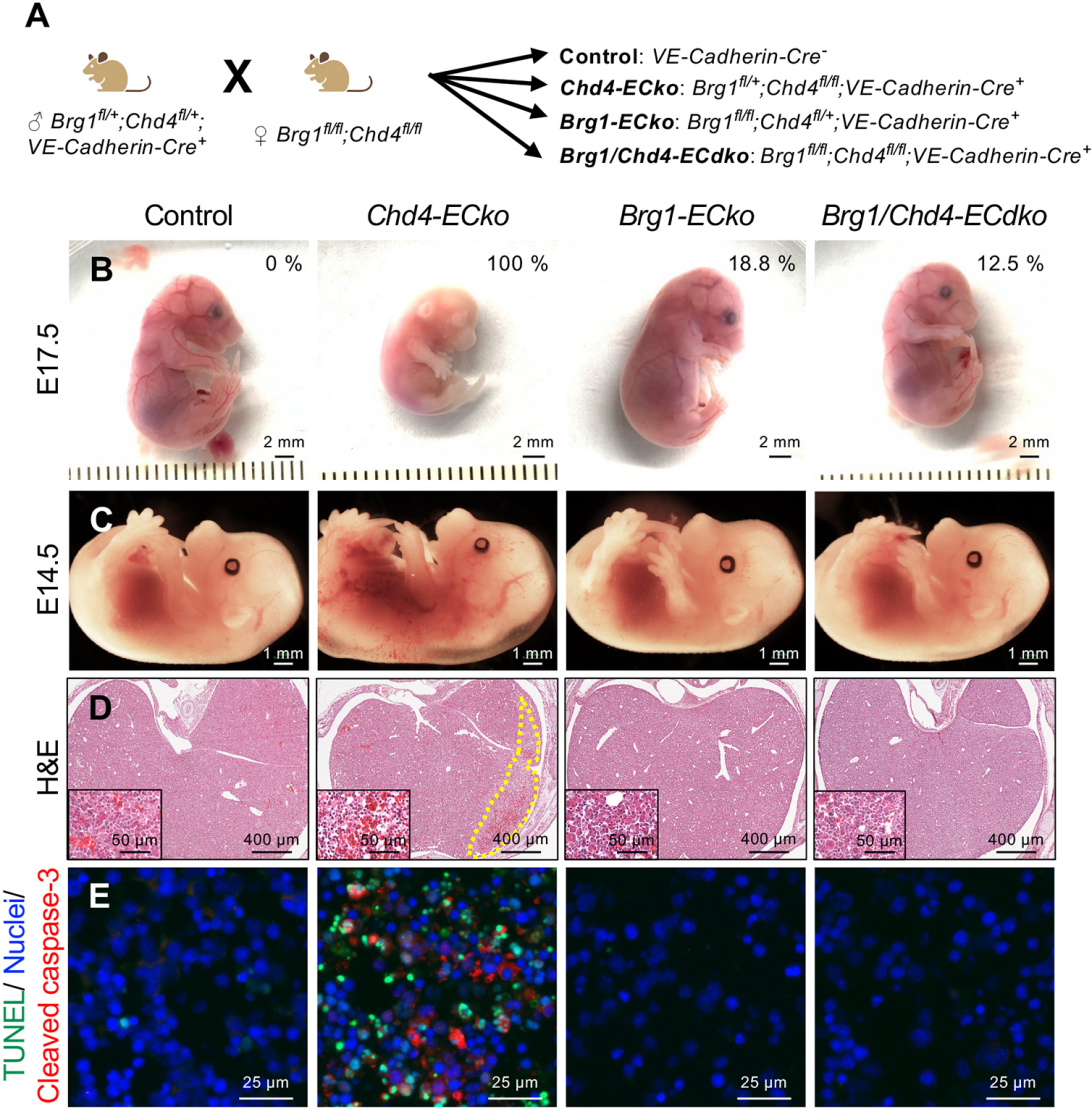
**Simultaneous deletion of endothelial *Brg1* and *Chd4* rescues the erythrocyte accumulation and cell death observed in *Chd4-ECko* livers.** (A) Diagram illustrating the crosses used to create endothelial-specific *Chd4*, *Brg1* and *Brg1/Chd4* double mutant embryos. (B) Representative images and lethality rates of control, *Chd4-ECko*, *Brg1-ECko* and *Brg1/Chd4-ECdko* embryos at E17.5. *n*≥8 embryos for each genotype. (C) Representative images of control, *Chd4-ECko*, *Brg1-ECko* and *Brg1/Chd4-ECdko* embryos at E14.5. *n*≥27 embryos for each genotype. (D) Representative images of E14.5 control and mutant livers stained with H&E. The dotted outline indicates a site of liver degeneration and erythrocyte accumulation. Insets show magnified images. (E) Representative images of E14.5 control and mutant liver sections stained by TUNEL (green) and immunostained for cleaved caspase-3 (red). Nuclei were counterstained with Hoechst dye (blue). *n*≥4 embryos for each genotype.

To understand how endothelial *Brg1/Chd4* mutants escape the lethality that occurs in endothelial *Chd4* mutant embryos by E16.5 (Crosswhite et al., 2016; Wu et al., 2024), we generated littermate embryos with deletions of endothelial *Chd4*, *Brg1* or both *Brg1/Chd4* by crossing *Brg1^fl/fl^;Chd4^fl/fl^* mice with *Brg1^fl/+^;Chd4^fl/+^;VE-cadherin-Cre^+^* mice (Fig. 1A). At E17.5, we detected 100% lethality in *Brg1^fl/+^;Chd4^fl/fl^;VE-cadherin-Cre^+^* (*Chd4-ECko*) embryos, 18.8% lethality in *Brg1^fl/fl^;Chd4^fl/+^;VE-cadherin-Cre^+^* (*Brg1-ECko*) embryos, and 12.5% lethality in *Brg1^fl/fl^;Chd4^fl/fl^;VE-cadherin-Cre^+^* (*Brg1/Chd4-ECdko*) embryos (Fig. 1B). Note that the shorthand names *Chd4-ECko* and *Brg1-ECko* do not reflect the respective heterozygosity for *Brg1* and *Chd4* that exists in these mutants due to our breeding strategy. At E14.5, *Brg1/Chd4-ECdko* mutants appeared grossly comparable to control (Cre-negative) and *Brg1-ECko* embryos (Fig. 1C). Notably, the histological accumulation of erythrocytes we detected in E14.5 *Chd4-ECko* mutant livers, as previously reported (Crosswhite et al., 2016), was rescued in *Brg1/Chd4-ECdko* mutants (Fig. 1D). Furthermore, we detected widespread cellular apoptosis in E14.5 *Chd4-ECko* livers by terminal deoxynucleotidyl transferase dUTP nick end labeling (TUNEL) staining and cleaved caspase-3 immunostaining, which were dramatically reduced in *Brg1/Chd4-ECdko* mutants (Fig. 1E). Therefore, the lethal liver phenotypes detected in *Chd4-ECko* mutants by E17.5 were rescued in *Brg1/Chd4-ECdko* mutants.

Murine fetal livers become the predominant hematopoietic organ after E11.0 (Soares-da-Silva et al., 2020), which coincides with the time that *Chd4-ECko* embryos begin developing lethal liver phenotypes. Indeed, prior to E14.5, fetal livers are mostly composed of erythrocytes and hematopoietic cells rather than hepatocytes (Gao et al., 2022; Mesquita Peixoto et al., 2025). Since some blood cells in E14.5 livers display *VE-cadherin-Cre* activity (Alva et al., 2006), we sought to rule out the possibility that the phenotypes we detected in *Chd4-ECko* livers resulted from *Chd4* deletion in the blood cell lineage. To investigate this, we crossed *Chd4-*flox mice onto the *Vav-Cre* transgenic line, which targets the hematopoietic lineage (Georgiades et al., 2002), and assessed embryonic lethality at E18.5. We found that *Chd4^fl/fl^;Vav-Cre^+^* embryos survived until E18.5 without evidence of mid-gestational lethality (Fig. S1). These results suggest that the lethal liver phenotypes seen in *Chd4-ECko* embryos are likely to be caused by *Chd4* deletion in ECs and not in hematopoietic cells.

### Plasmin deficiency does not rescue *Chd4-ECko* lethality as efficiently as endothelial *Brg1* deletion

**Fig. 2.**
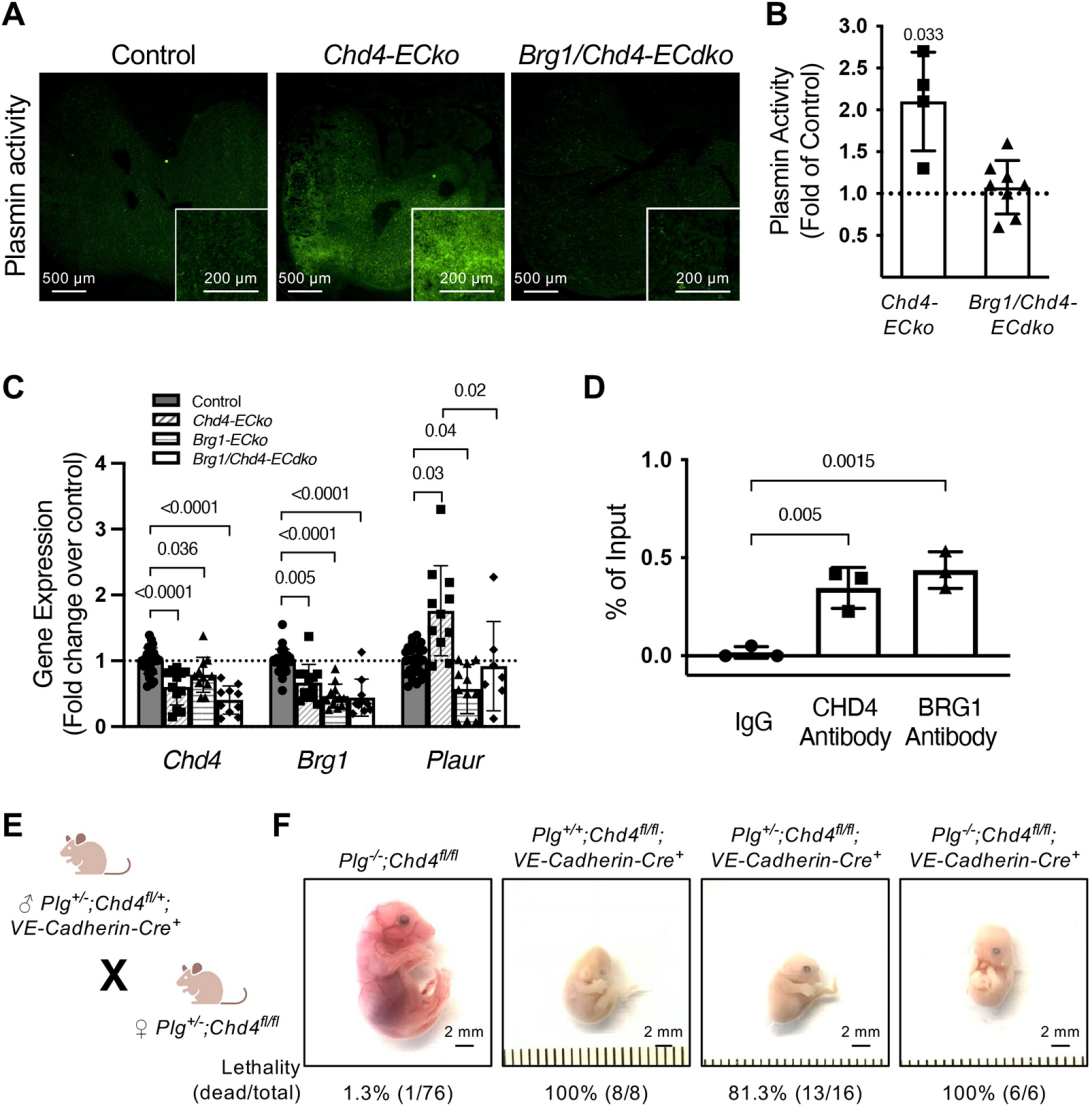
**Plasmin activation observed in *Chd4-ECko* livers is rescued in *Brg1/Chd4-ECdko* embryos, but genetic plasminogen reduction does not rescue *Chd4-ECko* lethality efficiently.** (A) Representative images of plasmin activity assessed by *in situ* zymography on unfixed E14.5 control and mutant liver sections. Insets show magnified images. (B) Mean fluorescence intensity from the *in situ* zymography plasmin activity assay, as shown in A. Each symbol represents the average intensity from four different regions analyzed within a liver section from a separate embryo. The dotted line represents the relative plasmin activity of liver sections from littermate controls. *n*≥4 embryos per genotype. (C) qRT-PCR analysis of *Chd4*, *Brg1* and *Plaur* gene transcripts in liver sinusoidal ECs isolated from E12.5 control and mutant embryos. Each symbol represents transcripts from separate embryos. The dotted line represents the relative expression of controls. *n*≥7 embryos per genotype, represented as individual symbols. (D) Enrichment of CHD4 and BRG1 at the *Plaur* gene loci was assessed by ChIP-qPCR assays in the MS1 murine EC line. *n*=3 independent immunoprecipitations (represented by symbols). (E) A schematic representation of the crosses performed to generate plasminogen (*Plg*)-deficient endothelial *Chd4* mutant embryos. (F) Representative images and lethality rates of the indicated mutant embryos at E17.5. Data are represented as mean (±s.d.). One-sample, two-tailed *t*-tests were used for the analysis in B. For C and D, ordinary one-way ANOVA with Dunnett's multiple comparisons post-hoc test or a Kruskal–Wallis test with Dunn's multiple comparisons post-hoc test was utilized when the results exhibited a nonparametric distribution.

We previously reported that deleting *Chd4* in embryonic ECs upregulates transcription of *Plaur*, thereby facilitating excessive plasmin activation, ECM degradation and liver degeneration (Crosswhite et al., 2016). Therefore, we sought to determine whether reduced plasmin activity contributed to the survival of *Brg1/Chd4-ECdko* embryos at E14.5. We first assessed plasmin activity by *in situ* zymography on fresh liver sections from E14.5 control and mutant embryos. We found that the increased plasmin activity in *Chd4-ECko* livers was rescued in *Brg1/Chd4-ECdko* livers (Fig. 2A,B). To determine whether this reduction in plasmin activity correlated with reduced *Plaur* transcripts in *Brg1/Chd4-ECdko* liver ECs, we isolated LYVE1^+^ liver sinusoidal ECs (LSECs) from control and mutant livers at E12.5, 2 days prior to the onset of liver degeneration in *Chd4-ECko* mutants, and analyzed the samples by quantitative reverse transcription PCR (qRT-PCR). The isolated LSEC samples showed enrichment for the LSEC marker *Stab2* but not for the hepatocyte marker *Afp* (Fig. S2A,B), and transcripts of *Chd4* and *Brg1* were reduced in LSECs from relevant mutants, as expected (Fig. 2C). *Plaur* transcripts were upregulated in LSECs from *Chd4* mutants, downregulated in LSECs from *Brg1* mutants, and neutralized in *Brg1/Chd4-ECdko* LSECs (Fig. 2C). We previously reported that *Plaur* is a direct gene target of CHD4 in a cultured murine extra-embryonic EC line (Ingram et al., 2013). We questioned whether *Plaur* is also a target of BRG1 in cultured ECs. To address this question, we performed ChIP followed by quantitative PCR (ChIP-qPCR) to determine whether BRG1 can interact with the *Plaur* gene promoter in the murine MS1 EC cell line. We found that BRG1 interacted with the same *Plaur* promoter region as CHD4 (−823 bp upstream of the transcription start site) (Fig. 2D). Together, these findings indicate that BRG1 and CHD4 directly and antagonistically impact *Plaur* transcription in LSECs to normalize embryonic hepatic plasmin activity.

We suspected that elevated plasmin was the primary contributor to the lethal liver phenotypes in *Chd4-ECko* livers, based on the rescue of plasmin activity, *Plaur* transcription, liver phenotypes, and lethality in *Brg1/Chd4-ECdko* mutants. Therefore, we predicted that genetic reduction of plasminogen (*Plg*), the zymogen of plasmin, in *Chd4-ECko* embryos would phenocopy the rescue seen in *Brg1/Chd4-ECdko* mutants. To test this, we crossed *Plg*^+/−^*;Chd4^fl/+^;VE-cadherin-Cre^+^* mice with *Plg*^+/−^*;Chd4^fl/fl^* mice to generate *Chd4-ECko* embryos with different doses of global plasminogen deficiency (Fig. 2E). To our surprise, lethality was not rescued in either *Plg*^+/−^*;Chd4^fl/fl^;VE-cadherin-Cre^+^* or *Plg*^−/−^*;Chd4^fl/fl^;VE-cadherin-Cre^+^* embryos comparably to the rescue we saw in *Brg1/Chd4-ECdko* embryos at E17.5. Thirteen out of 16 *Plg^+/−^;Chd4^fl/fl^;VE-cadherin-Cre^+^* embryos we dissected displayed advanced resorption at E17.5 (81.3% lethality), while each of the six *Plg^−/−^;Chd4^fl/fl^;VE-cadherin-Cre^+^* embryos we dissected was resorbed (100% lethality) (Fig. 2F). As a reminder, this was much higher than the 12.5% lethality we saw in E17.5 in *Brg1/Chd4-ECdko* mutants (Fig. 1B).

Coagulation abnormalities can be transferred bidirectionally between mothers and fetuses (Bishop et al., 1971). Therefore, we wondered whether maternal plasminogen contributed to the lethality of *Chd4-ECko* embryos with elevated uPA expression. We tested this hypothesis by generating mutant embryos from *Plg^−/−^;Chd4^fl/fl^;VE-cadherin-Cre^+^* dams (Fig. S3A). We did see increased embryonic rescue at E17.5, with *Plg*^+/−^*;Chd4^fl/fl^;VE-cadherin-Cre^+^* lethality dropping to 50% and *Plg*^−/−^*;Chd4^fl/fl^;VE-cadherin-Cre^+^* lethality dropping to 70.8% (Fig. S3B). However, this increased survival still did not match the rescue seen in *Brg1/Chd4-ECdko* embryos, indicating that factors other than elevated plasmin likely contribute to lethal liver phenotypes in *Chd4-ECko* embryos.

### Transcriptomics reveal sterile inflammation in *Chd4-ECko* livers

**Fig. 3.**
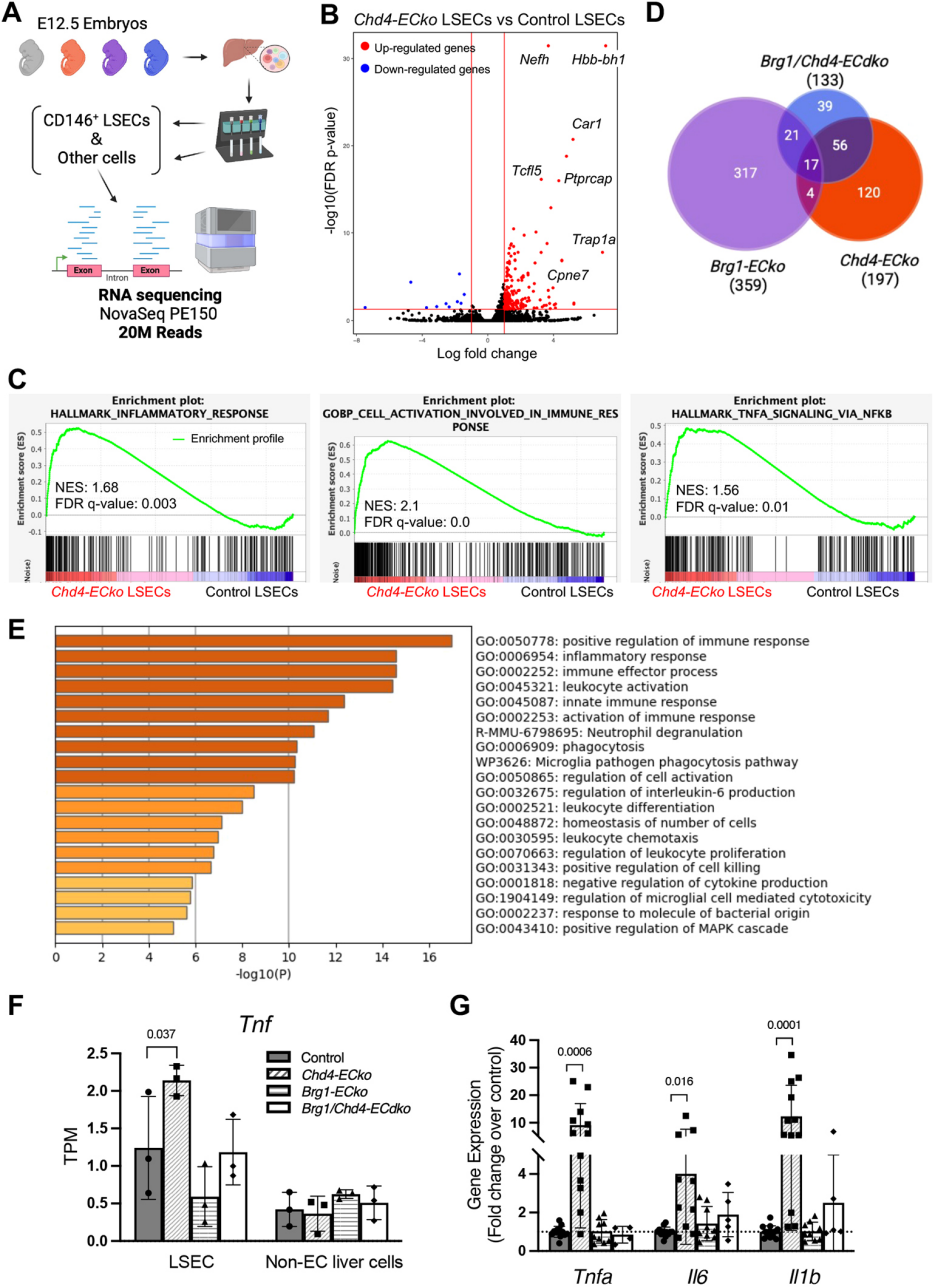
**Transcriptomics indicate that *Chd4-ECko* LSECs are inflamed.** (A) A schematic illustrating LSEC isolation and RNA sequencing. (B) Volcano plot of all genes in *Chd4-ECko* LSECs compared to control LSECs. DEGs were defined as having more than a twofold change in expression in *Chd4-ECko* LSECs relative to control LSECs, with an adjusted *P*<0.05. Significantly upregulated genes are shown in red, and significantly downregulated genes in blue. (C) GSEA revealed ‘inflammatory response’, ‘cell activation involved in immune response’ and ‘TNFα signaling via NFκB’ gene sets in *Chd4-ECko* LSECs. (D) Venn diagram illustrating the number of DEGs in *Chd4-ECko*, *Brg1-ECko* and *Brg1/Chd4-ECdko* LSECs. (E) GO and Kyoto Encyclopedia of Genes and Genomes (KEGG) pathway enrichment analyses were performed on the 120 DEGs that were exclusively altered in *Chd4-ECko* LSECs. (F) *Tnf* transcripts in LSECs and non-EC liver cells from E12.5 control and mutants, gleaned from RNA-Seq data. TPM, transcripts per million mapped reads. *n*=3 embryos per genotype, represented as individual symbols. (G) qRT-PCR analysis of *Tnf*, *Il6* and *Il1b* gene transcripts in E14.5 control and mutant livers. The dotted line shows the relative expression of controls. *n*≥4 embryos, represented as individual symbols. Data are represented as mean (±s.d.). An ordinary one-way ANOVA with Dunnett's multiple comparisons post-hoc test was used for the analysis in F. For G, a Kruskal–Wallis test with Dunn's multiple comparisons post-hoc test was used due to the nonparametric distribution of the results.

To identify other potential contributors to *Chd4-ECko* lethal liver phenotypes, we performed RNA sequencing (RNA-Seq) on CD146 (MCAM)^+^ LSECs and non-LSEC liver cells isolated from E12.5 control and mutant livers 2 days before liver degeneration and lethality occurred (Fig. 3A). Principal component analysis and clustering of EC marker genes showed distinct gene expression between LSEC and non-EC groups (Fig. S4A,B). Because the CHD4-containing NuRD complex also contains histone deacetylases and methyl-CpG-binding domain proteins, it is typically considered a repressive complex (Curtis et al., 2012). Concordantly, we found that most differentially expressed genes (DEGs: log_2_ fold change>1, adjusted *P*<0.05) in *Chd4-ECko* LSECs were upregulated compared to control LSECs (Fig. 3B, Fig. S4C). Employing gene set enrichment analysis (GSEA) for control and *Chd4-ECko* LSECs, we found enrichment of genes associated with the terms ‘inflammatory response’, ‘cell activation involved in immune response’ and ‘TNFα signaling via NFκB’ in *Chd4-ECko* LSECs (Fig. 3C). To identify genes and pathways contributing to *Chd4-ECko* liver phenotypes, we identified 120 DEGs that were exclusively changed in *Chd4-ECko* LSECs (Fig. 3D). A Gene Ontology (GO) biological process analysis of these 120 DEGs showed that they were associated with hallmarks of inflammation, including inflammatory and immune responses and cell activation (Fig. 3E). Since TNFα signaling can mediate liver degeneration in a similar manner to what we observed in *Chd4-ECko* mutants (Doi et al., 1999), we analyzed *Tnf* transcripts from our RNA-Seq data and found them to be significantly upregulated in *Chd4-ECko* LSECs (Fig. 3F). Meanwhile, *Rela* (the gene name of NFκB p65), the effector of the NFκB pathway that counterbalances TNFα signaling to achieve normal liver development, was unaffected in all mutants (Fig. S5). To analyze sterile inflammation in *Chd4-ECko* livers more directly, we next used qRT-PCR to assess transcripts of cytokine genes that were upregulated in *Chd4-ECko* LSECs by RNA-Seq. We found that *Tnf*, *Il6* and *Il1b* transcripts were significantly increased in *Chd4-ECko* livers and were normalized in *Brg1/Chd4-ECdko* livers at E14.5 (Fig. 3G). Additionally, when we exploited our RNA-Seq data to examine transcripts associated with the plasminogen activation system, we found that *Plaur* was the only gene that was significantly induced in *Chd4-ECko* LSECs at E12.5 (Fig. S4D). These results indicate that, in addition to promoting plasmin activation, the livers of *Chd4-ECko* embryos display evidence of heightened sterile inflammation.

**Fig. 4.**
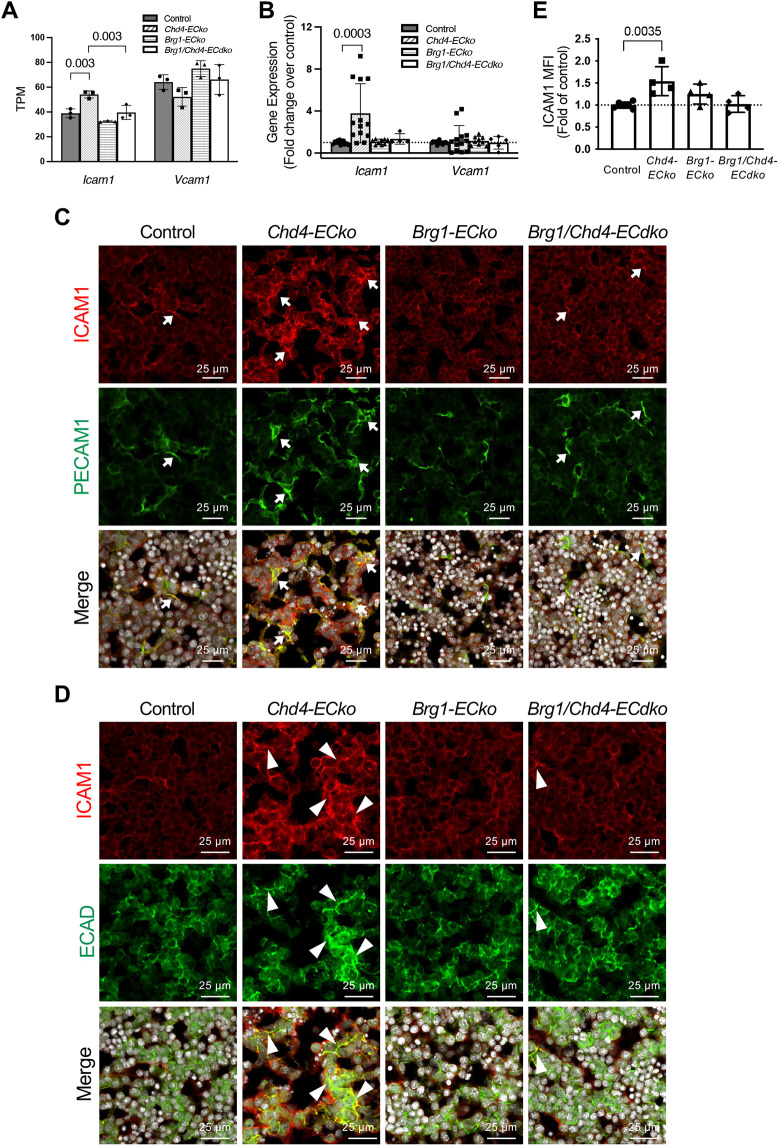
**ICAM1 expression is increased in endothelial cells and hepatoblasts of *Chd4-ECko* livers.** (A) Transcripts of *Icam1* and *Vacm1* in E12.5 control and mutant LSECs, gleaned from RNA-Seq data. Each symbol represents data from a separate embryo. TPM, transcripts per million mapped reads. (B) qRT-PCR analysis of *Icam1* and *Vcam1* gene transcripts in E14.5 control and mutant livers. The dotted line shows the relative expression of controls. *n*≥4 embryos (shown as individual symbols). (C) Representative images of immunostaining for ICAM1 (red) and the EC marker PECAM1 (green) in E14.5 control and mutant livers. The merged images include a nuclear stain (Hoechst) displayed in gray. Arrows indicate cells that express both ICAM1 and PECAM1. *n*≥4 embryos for each genotype. (D) Representative images of immunostaining for ICAM1 (red) and the hepatoblast marker ECAD (CDH1; green) are shown in E14.5 control and mutant livers. The merged images include a nuclear stain displayed in gray. Arrowheads indicate cells expressing ICAM1 and ECAD. *n*≥2 embryos for each genotype. (E) Quantification of total ICAM1 protein levels based on mean fluorescence intensity (MFI) in immunostained E14.5 control and mutant livers. *n*≥4 embryos (shown as individual symbols). The dotted line shows the relative expression of controls. Data are represented as mean (±s.d.). Ordinary one-way ANOVA with Dunnett's multiple comparisons post-hoc tests were used for analysis in A and E. For B, a Kruskal–Wallis test with Dunn's multiple comparisons post-hoc test was used due to nonparametric data distribution.

To assess whether the microenvironment of *Chd4-ECko* livers was altered under sterile inflammation, we characterized cellular components by immunostaining for various markers in E12.5 to E14.5 livers. These markers included LYVE1 for liver sinusoids; Von Willebrand factor (VWF) and PECAM1 for ECs (VWF can be found on some ECs); PROX1 and E-cadherin (ECAD) for hepatocytes; CD41 and VWF for platelets and megakaryocytes; CD45 (PTPRC) for leukocytes; CD68 and F4/80 (ADGRE1) for macrophages; myeloperoxidase (MPO) for neutrophils; TER119 (LY76) for erythroid cells; and α-smooth muscle actin (SMA; ACTA2) for pericytes and smooth muscle cells. Across all the images we examined, no obvious cell type-specific differences in these markers were observed between control and mutant livers (Fig. 4D, Fig. S6A-G).

### Expression of the endothelial adhesion molecule ICAM1 is elevated in *Chd4-ECko* hepatic ECs

We next sought to understand endothelial factors contributing to inflammation in *Chd4-ECko* livers. Since BRG1 can bind to the *Tnf* gene locus to induce its expression in mouse kidneys after renal ischemic injury (Naito et al., 2009), we initially tried to examine BRG1 and CHD4 binding to the *Tnf* locus in control and mutant embryonic LSECs using ChIP-qPCR. However, we were unable to collect enough LSECs from embryonic livers to perform immunoprecipitation. Instead, we investigated whether *Tnf* is a direct target of CHD4 or BRG1 in MS1 ECs using ChIP-qPCR. We found that BRG1 was enriched at the *Tnf* locus under basal conditions, but CHD4 was not enriched there (Fig. S7A,B). Therefore, although *Tnf* was upregulated in total livers and in LSECs of *Chd4-ECko* embryos (Fig. 3F,G), we continued our search for inflammatory factors that might be regulated directly by CHD4 in ECs.

Our RNA-Seq data revealed that transcripts for the adhesion molecule *Icam1* were upregulated in E12.5 *Chd4-ECko* LSECs and normalized in *Brg1/Chd4-ECdko* LSECs (Fig. 4A). This seemed relevant to *Chd4-ECko* hepatic phenotypes because endothelial ICAM1 induction causes leukocyte infiltration and cytokine production (Dragoni et al., 2017; Guerra-Espinosa et al., 2024) and can impair vascular barrier function (Clark et al., 2007; Sumagin et al., 2008). Notably, transcripts for another endothelial adhesion molecule, VCAM1, were not upregulated in *Chd4-ECko* LSECs (Fig. 4A). We next confirmed by qRT-PCR that *Icam1* transcripts were also elevated in *Chd4-ECko* livers at E14.5 (Fig. 4B). We also assessed ICAM1 protein expression by immunostaining and found it to be increased on both ECs and hepatoblasts of *Chd4-ECko* livers at E14.5 (Fig. 4C-E). This result was consistent with reports that cytokines can induce ICAM1 expression in hepatocytes (Satoh et al., 1994; Sano et al., 1999). Altogether, unbiased transcriptomic data led to our discovery that *Icam1* is transcriptionally upregulated in *Chd4-ECko* embryonic livers prior to their lethal deterioration.

### CHD4 and BRG1 antagonistically regulate *Icam1* transcripts in ECs

**Fig. 5.**
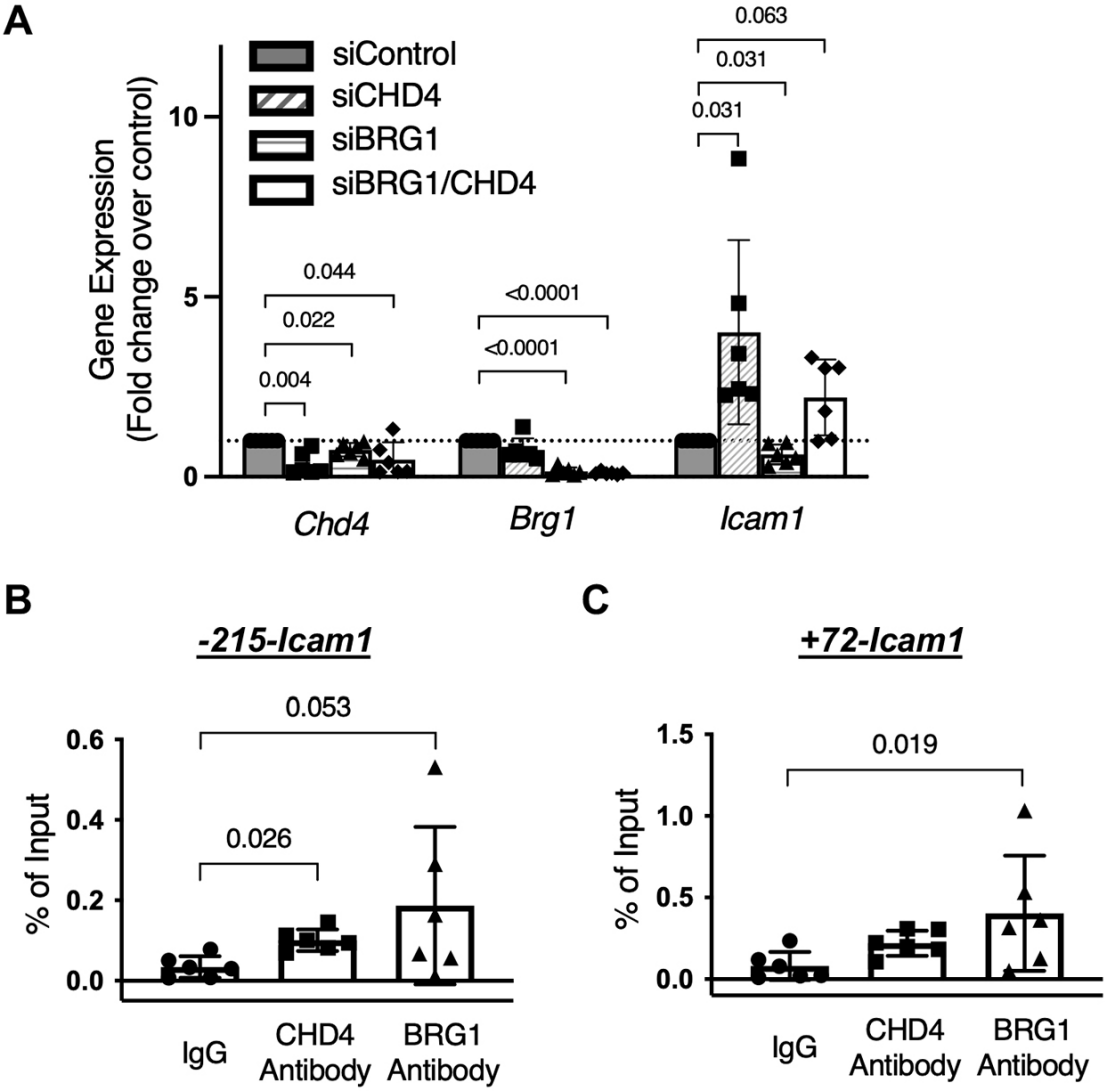
**CHD4 and BRG1 antagonistically regulate *Icam1* transcription in cultured murine ECs.** (A) qRT-PCR analysis of *Chd4*, *Brg1* and *Icam1* gene transcripts after CHD4 and/or BRG1 knockdown in MS1 ECs (*n*=6 independent experiments). The dotted line shows the relative expression in cells treated with control siRNA. (B,C) ChIP-qPCR was used to determine the enrichment of CHD4 and BRG1 at regions 215 bp upstream (−215) and 72 bp downstream (+72) of the *Icam1* transcription start site in MS1 ECs. *n*=6 independent immunoprecipitations (individual symbols). Data are represented as mean (±s.d.). One-sample, two-tailed *t*-tests were used for the analysis in A. For B and C, a Kruskal–Wallis test with Dunn's multiple comparisons post-hoc test was used due to nonparametric data distribution.

BRG has been shown to bind the *ICAM1* gene promoter and promote its transcription in human umbilical vein ECs (Fang et al., 2013). To investigate whether *Icam1* is also a target gene of BRG1 and CHD4 in murine ECs, we assessed *Icam1* transcripts after knocking down CHD4 and/or BRG1 in MS1 cells. We found that *Icam1* expression was upregulated upon CHD4 knockdown, downregulated upon BRG1 knockdown, and normalized when both chromatin-remodeling enzymes were knocked down simultaneously (Fig. 5A). We next performed ChIP-qPCR on MS1 cells to determine whether CHD4 and BRG1 could interact directly with *Icam1* regulatory elements. We found enrichment for both CHD4 and BRG1 in the promoter region of *Icam1* (−215 bp upstream of the transcription start site) (Fig. 5B). We also detected BRG1 enrichment at an intronic region (+72 bp downstream of the transcription start site) of the *Icam1* gene (Fig. 5C). Collectively, these findings demonstrate that CHD4 and BRG1 work antagonistically in regulating *Icam1* expression in ECs.

### Carprofen treatment combined with plasminogen deficiency rescues *Chd4-ECko* lethality

**Fig. 6.**
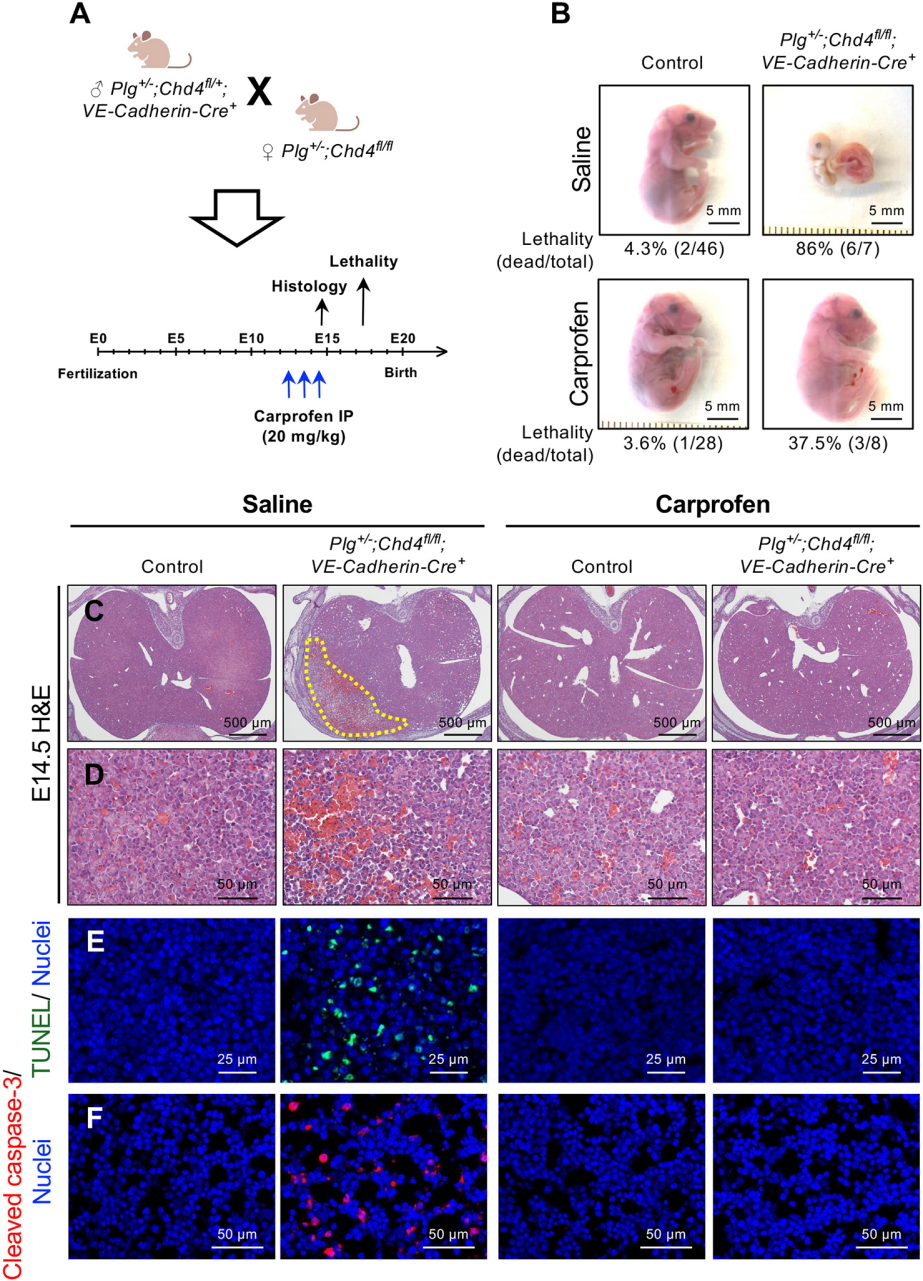
**Combining anti-inflammatory carprofen treatment and genetic plasminogen deficiency rescues *Chd4-ECko* lethality efficiently.** (A) Schematic of the experimental design for the combination treatment of carprofen (20 mg/kg) and *Plg* deficiency in *Chd4^fl/fl^;VE-cadherin-Cre^+^* mutants. (B) Representative images and lethality rates of control and *Plg^+/−^;Chd4^fl/fl^;VE-cadherin-Cre^+^* embryos after saline and carprofen treatment at E17.5. (C,D) H&E staining of saline- and carprofen-treated control and *Plg^+/−^;Chd4^fl/fl^;VE-cadherin-Cre^+^* livers at E14.5. The dotted outline indicates liver deterioration and erythrocyte accumulation. (E) Representative images of TUNEL staining (green) in saline- and carprofen-treated control and *Plg^+/−^;Chd4^fl/fl^;VE-cadherin-Cre^+^* liver sections at E14.5. *n*≥2 embryos for each genotype. (F) Representative images of immunostaining for cleaved caspase-3 (red) in saline- and carprofen-treated control and *Plg^+/−^;Chd4^fl/fl^;VE-cadherin-Cre^+^* liver sections at E14.5. *n*≥2 embryos for each genotype. Nuclei were counterstained with Hoechst dye (blue).

Since we observed evidence of excessive plasmin activation and inflammation in *Chd4* mutant livers, we questioned whether genetic deletion of *Plg* combined with an anti-inflammatory treatment could rescue *Chd4-ECko* liver phenotypes and lethality. We performed timed matings to generate *Chd4-ECko* embryos with *Plg* deficiency, as before, and treated pregnant dams with saline (vehicle control) or the nonsteroidal anti-inflammatory drug carprofen (20 mg/kg) for three consecutive days starting at E12.5 (Fig. 6A). We then assessed embryonic lethality in *Plg*^+/−^*;Chd4^fl/fl^;VE-cadherin-Cre^+^* embryos at E17.5 since we had observed a slight improvement in *Plg*^+/−^*;Chd4^fl/fl^;VE-cadherin-Cre^+^* embryonic survival at baseline (81.3% lethality; Fig. 2F). We found the lethality of *Plg*^+/−^*;Chd4^fl/fl^;VE-cadherin-Cre^+^* embryos dropped from 86% (saline treated) to 37.5% after carprofen treatment (Fig. 6B). We also assessed embryos histologically at E14.5 and found erythrocyte accumulation in saline-treated *Plg*^+/−^*;Chd4^fl/fl^;VE-cadherin-Cre^+^* livers was eliminated after carprofen treatment (Fig. 6C,D). Additionally, the widespread apoptosis that we detected in saline-treated *Plg*^+/−^*;Chd4^fl/fl^;VE-cadherin-Cre^+^* livers was diminished after carprofen treatment (Fig. 6E,F). Notably, treating *Chd4* mutants with carprofen alone still resulted in 89% lethality (Table 1), demonstrating that the improved survival in carprofen-treated *Plg^+/−^;Chd4^fl/fl^;VE-cadherin-Cre^+^* embryos was not achieved by the nonsteroidal anti-inflammatory drug alone. Therefore, both plasmin activation and sterile inflammation synergistically contributed to *Chd4-ECko* embryonic liver deterioration and lethality.

**Table 1.** Lethality of carprofen-treated embryos at E17.5 (*Chd4^fl/fl^×Chd4^fl/+^;VE-cadherin-Cre^+^*)

Genotype	Saline	Carprofen
Control	0/27 (0%)	0/35 (0%)
*Chd4^fl/fl^;VE-cadherin-Cre^+^*	11/11 (100%)	8/9 (89%)

Lethality is defined as the number of dead embryos out of the total number of embryos (also expressed as a percentage).

*P*>0.9999 examined by Fisher's exact test.

## DISCUSSION

Embryonic vessels expand to support organ growth during fetal development. Therefore, vascular defects during development can lead to cell death, organ degeneration and embryonic lethality. This study demonstrates that the endothelial chromatin remodelers CHD4 and BRG1 have antagonistic effects on the activity of the protease plasmin and on sterile inflammation in fetal livers. We found that treatment with the anti-inflammatory drug carprofen, combined with plasminogen deficiency, successfully prevented liver degeneration and embryonic lethality. This indicates that ECs have the capacity to play a pro-inflammatory role in embryonic murine livers and highlights the need for their tight regulation to ensure normal liver growth.

### Increased plasmin activity only partially contributes to *Chd4-ECko* lethality

When we began this study, we assumed that excessive plasmin activity was the primary driver of embryonic *Chd4-ECko* liver deterioration and lethality. This assumption was based on our previous report that genetic deletion of the gene encoding the plasmin activator uPA delays embryonic death in endothelial *Chd4* mutants (Crosswhite et al., 2016). We hypothesized that loss of the plasmin precursor, plasminogen (*Plg*), would more effectively prevent death of *Chd4-ECko* mutants. However, *Plg* deficiency provided minimal rescue of *Chd4* lethality, with only three out of 16 *Plg^+/−^;Chd4^fl/fl^;VE-cadherin-Cre^+^* mutants surviving to E17.5 (81.3% lethality; Fig. 2F). Interestingly, we observed a greater rescue effect when *Plg*-deficient endothelial *Chd4* mutants were generated from *Plg^−/−^* dams (Fig. S2B). These findings indicate a potential role for maternal plasminogen in influencing the liver phenotypes seen in *Chd4-ECko* mutants. However, further investigation is needed to determine how maternal plasminogen crosses the placenta into fetal circulation. Notably, *Brg1/Chd4-ECdko* mutants exhibited the most significant rescue of *Chd4-ECko* lethality among all the rescue effects we observed, although admittedly not all *Brg1/Chd4-ECdko* mutants survived until E17.5 (one out of eight embryos was dead upon dissection; Fig. 1B). The reason for this incomplete rescue and for the 18.8% lethality of *Brg1-ECko* embryos we likewise detected at E17.5 (Fig. 1B) is still unclear. Nevertheless, our observation that more *Brg1/Chd4-ECdko* mutants survived to E17.5 than did *Plg^+/−^;Chd4^fl/fl^;VE-cadherin-Cre^+^* mutants indicates that endothelial BRG1 and CHD4 antagonistically impact more than just plasmin activation in embryonic livers.

### Potential roles of plasmin and uPAR in promoting inflammation

While the role of the plasminogen activation system in fibrinolysis is well-recognized, there is increasing evidence that it also serves as a potent regulator of additional processes, including inflammation (Smith and Marshall, 2010; Doeuvre et al., 2010; Syrovets et al., 2012; Draxler et al., 2017; Hamada et al., 2024). For example, plasmin and uPAR promote inflammation across multiple cell types by facilitating cellular activation, cytokine production, chemotaxis, cell death and cyclooxygenase 2 (COX2; PTGS2) production (Chang et al., 1993; Gyetko et al., 2000, 2001; Syrovets et al., 2001; Burysek et al., 2002; Rossignol et al., 2006; Doeuvre et al., 2010; Syrovets et al., 2012; Draxler et al., 2017; Hamada et al., 2024). Additionally, plasmin has been shown to induce ICAM1 expression in human umbilical vein ECs (Li et al., 2013), and uPAR is required for leukocyte recruitment to the lung in response to *Pseudomonas aeruginosa* infection (Gyetko et al., 2000). In the case of uPAR, this receptor can coordinate cellular signaling through interactions with vitronectin, integrins and growth factor receptors (Blasi and Carmeliet, 2002; Smith and Marshall, 2010; Hamada et al., 2024), so it may mediate mechanisms in addition to plasmin activation to promote inflammation. Future experiments will be needed to determine whether the elevated uPAR expression in *Chd4-ECko* mutants contributes to the sterile inflammation and liver deterioration that is not effectively rescued by *Plg* deletion alone.

### Roles of endothelial BRG1 and CHD4 in mediating sterile inflammation

Previous reports indicate that chromatin-remodeling enzymes interact to regulate inflammatory gene expression. For example, BRG1 and CHD4 antagonistically regulate the transcription of inflammatory genes in a cultured macrophage line upon lipopolysaccharide stimulation (Ramirez-Carrozzi et al., 2006). Moreover, BRG1 promotes TNFα expression following liver and kidney injury (Naito et al., 2009; Wang et al., 2023). TNFα is known to cause apoptosis and liver degeneration in mouse embryos with impaired NFκB signaling (Doi et al., 1999; Li et al., 1999; Rudolph et al., 2000). Consistent with this evidence that unchecked TNFα signaling is detrimental to embryonic liver development, *Chd4-ECko* livers exhibited increased *Tnf* transcripts and activated TNFα signaling (Fig. 3C,F,G). However, we did not observe CHD4 interacting with the *Tnf* gene locus in the murine ECs we analyzed, so we suspect that *Tnf* is not a direct CHD4 target gene in ECs and that elevated *Tnf* expression in *Chd4-ECko* livers is an indirect consequence of *Chd4* deletion.

Meanwhile, BRG1 and BRM (SMARCA2), alternative remodeling enzymes belonging to SWI/SNF complexes, mediate the expression of ICAM1 and VCAM1 in ECs during the progression of atherosclerosis (Fang et al., 2013). BRG1 is reported to promote *Icam1* transcription through binding its intronic regions in both cultured ECs and glioma cell lines (Fang et al., 2013; Kesanakurti et al., 2013). This is consistent with our observations that BRG1 is enriched in the intronic region of *Icam1* in murine MS1 ECs (Fig. 5C). In this study, we provide additional evidence that CHD4 works antagonistically with BRG1 in regulating *Icam1* expression in ECs (Fig. 5A). We speculate that elevated ICAM1 expression in *Chd4-ECko* ECs may tip the hepatic environment toward detrimental sterile inflammation, thereby disrupting the normal organogenesis mediated by TNFα-triggered NFκB signaling (Li et al., 1999; Doi et al., 1999; Rudolph et al., 2000; Rosenfeld et al., 2000). Nevertheless, we acknowledge that additional genetic deletion of *Icam1* or *Tnf* in *Chd4-ECko* mutants would be required to clarify the respective contributions of ICAM1 and TNF to liver degeneration and lethality in *Chd4-ECko* embryos.

Finally, the impact of sterile inflammation on hepatic vascular phenotypes and hematopoiesis is worth considering in this study. Inflammatory cytokines and ICAM1 are known to impair EC barrier function and mediate vascular permeability (Wójciak-Stothard et al., 1998; Bui et al., 2020). We noticed that sinusoidal capillaries in *Chd4-ECko* livers exhibited a larger capillary diameter than those of littermate controls (Fig. S6C). However, this phenotype was also evident in *Brg1/Chd4-ECdko* livers (Fig. S6C), despite the concomitant reduction in inflammatory cytokines and endothelial ICAM1 expression in these double mutants (Figs 3F,G, 4A-C). Therefore, although *Chd4-ECko* livers exhibit dilated sinusoids, this dilation is not likely to be caused by sterile inflammation or to be associated with erythrocyte accumulation or liver degeneration, which are rescued in *Brg1/Chd4-ECdko* livers that likewise have dilated sinusoids. As for hematopoiesis, the process appears to be normal in p65 and IKKβ knockout livers with compromised NFκB signaling and aberrant TNFα signaling (Beg et al., 1995; Li et al., 1999). However, there is abundant evidence that ICAM1 and inflammatory cytokines may influence hematopoiesis in other contexts (Orelio et al., 2009; Li et al., 2014; Espin-Palazon et al., 2014; Liu et al., 2018; Collins et al., 2021). Therefore, future studies could address whether hematopoiesis is dysregulated in *Chd4-ECko* mutant livers prior to lethality.

Overall, this study reveals that ECs play a crucial role in embryonic liver development through their precise transcriptional regulation of plasmin activation and sterile inflammation. These findings underscore the significant functions of ECs in ensuring proper liver development and provide insights about the synergistic and detrimental effects of plasmin and inflammation in the liver.

### Limitations of study

To obtain practical odds of recovering single- and double-mutant embryos within the same litter, we used *Brg1^fl/fl^;Chd4^fl/fl^* females, rather than *Brg1^fl/+^;Chd4^fl/+^* females, for timed matings (Fig. 1A). We acknowledge that the single mutants generated with this strategy are heterozygous for the other chromatin remodeler. However, because we still observed 100% lethality in *Chd4-ECko* mutants, these data indicate that loss of a single *Brg1* allele cannot rescue *Chd4*-associated lethality. In addition, we did not always obtain Cre-negative littermate *Brg1^fl/fl^;Chd4^fl/fl^* control embryos alongside single-mutant or double-mutant embryos. Therefore, we also used embryos lacking *Cre* but carrying at least one floxed allele for both *Brg1* and *Chd4* as controls in this study. Finally, the embryonic liver is the major hematopoietic organ at the time the *Chd4-ECko* phenotype emerges, and at this stage it contains a heterogeneous mixture of immune cells that contribute to normal liver development (Si-Tayeb et al., 2010; Rezvani et al., 2026). Although we did not observe changes in the immune cell markers we analyzed in *Chd4* mutants, we cannot exclude the possibility that a subset of immune cells, including specific macrophages, contributes to the rescue observed in *Brg1/Chd4-ECdko* livers.

## MATERIALS AND METHODS

### Mouse lines and embryo dissections

*Brg1-floxed* mice (Sumi-Ichinose et al., 1997), *Chd4-floxed* mice (Williams et al., 2004), *VE-Cadherin-Cre* transgenic mice (Alva et al., 2006), *Plg*-deficient mice (Ploplis et al., 1995) and *Vav-iCre* transgenic mice (de Boer et al., 2003) were generated and genotyped as described (Griffin et al., 2008; Curtis and Griffin, 2012; Wiley et al., 2015; Gao et al., 2018; Colijn et al., 2020). *Brg1^fl/fl^;Chd4^fl/fl^* mice were bred to *Brg1^fl/+^;Chd4^fl/+^;VE-Cadherin-Cre* mice to generate *Brg1-ECko*, *Chd4-ECko* and *Brg1/Chd4-ECdko* mutants, which were maintained on a mixed genetic background. *Chd4^fl/fl^;Plg^+l−^* or *Chd4^fl/fl^;Plg^−l−^* mice were bred to *Chd4^fl/+^;Plg^+l−^;VE-Cadherin-Cre* mice to generate *Plg*-deficient *Chd4* mutant embryos. Mice used for timed matings ranged from 8 weeks to 1 year of age. Noon on the day of vaginal plug detection was designated as E0.5. Embryonic yolk sacs were genotyped, and littermates that tested negative for Cre recombinase were used as controls to minimize potential genetic background effects. Saline or carprofen (Pivetal^®^ LEVAFEN™ injection, 20 mg/kg) was administered via intraperitoneal injection to the dams for three consecutive days starting from E12.5. Whole embryos were collected for histological analysis, and embryonic livers were isolated for the indicated experiments. Mouse embryo images were captured using a Nikon SMZ800 stereomicroscope and Nikon DS-Fi1 camera. All mice were fed with a standard diet (LabDiet 5053 - PicoLab^®^ Rodent Diet 20, composed of 20% protein and 4.5% fat) and were housed at the Oklahoma Medical Research Foundation (OMRF), which is an American Association for Accreditation of Laboratory Animal Care (AAALAC) accredited facility. Animal care was provided in accordance with the procedures outlined in the Guide for Care and Use of Laboratory Animal (National Academies Press; Washington, D.C.; 1996). All animal experiments were conducted under the approval of the OMRF Institutional Animal Care and Use Committee (IACUC, #24-27). Data from male and female embryos were pooled into one group for analysis since no obvious gender effects were observed. Experimental animals were selected based on genotype; the small numbers of experimental (i.e. mutant) animals within each litter excluded a need for randomization of this category.

### *In situ* zymography

Plasmin activity was assessed using the plasmin-specific fluorogenic substrate Boc-Glu-Lys-Lys-MCA (Peptide Institute Inc., 3105-v) as previously described (Gao et al., 2018). Freshly collected unfixed liver cryosections (12 μm) were obtained from E14.5 embryos. Sections were overlaid with *in situ* zymography solution containing 1% Ultra Pure LMP Agarose (Invitrogen, 16520), 0.4 mM Boc-Glu-Lys-Lys-MCA, and 1 U/ml human Glu-plasminogen (Sigma-Aldrich). Overlaid sections were coverslipped and incubated for 1 h at 37°C. To confirm that MCA cleavage was dependent on plasmin, plasminogen-free *in situ* zymography solution was used as a negative control. Substrate fluorescence was detected by an Eclipse Ti-E epifluorescence microscope (Nikon) and analyzed with Nikon Element (v4) or Fiji software.

### Histology and TUNEL assay

Embryos were fixed overnight at 4°C in 4% paraformaldehyde diluted in PBS. After fixation, samples were dehydrated and embedded in paraffin for sectioning. Paraffin sections (4 μm) were prepared for Hematoxylin and Eosin (H&E) staining. Brightfield images of these sections were captured using an Eclipse 80i microscope (Nikon) and a DS-Fi1 camera (Nikon). For TUNEL staining, paraffin sections or cryosections from E14.5 control and mutant livers were analyzed with the *In situ* Cell Death Detection Kit, fluorescein (Roche, 11684795910). In brief, paraffin sections were deparaffinized and rehydrated, while cryosections were rinsed in PBS to remove optimal cutting temperature compound (O.C.T.; Sakura Finetek). Sections were subjected to proteinase K digestion (20 μg/ml) for 30 min at 37°C, followed by incubation with the TUNEL reaction mixture (fluorescein-dUTP) for 1 h at 37°C, and then rinsed three times with PBS. After TUNEL labeling, performed immunostaining for cleaved caspase-3 and nuclear staining, if needed, were performed. Sections without TUNEL enzyme solution served as negative controls. Finally, slides were mounted using ProLong Gold mountant (Thermo Fisher Scientific, P36930). Images were acquired using an Eclipse Ti-E epifluorescence microscope or an AX-R confocal microscope (Nikon) and analyzed with Nikon Elements software (v4).

### Immunofluorescence

Embryos were dehydrated using gradients of sucrose at 10%, 15% and 20% after paraformaldehyde fixation. They were then incubated overnight at 4°C in a 1:1 mixture of 20% sucrose and O.C.T. The following morning, embryos were embedded in O.C.T., and 8 μm cryosections were slide mounted for immunofluorescence. Sections were permeabilized with 0.2% Triton X-100 in PBS for 15 min and subsequently blocked with 3% bovine serum albumin (BSA) in PBS. All sections were immunostained overnight at 4°C with primary antibodies diluted in 1% BSA in PBS. The primary antibodies used were: anti-cleaved caspase-3 (1:100; Cell Signaling Technology, 9661; RRID: AB_2341188), anti-PECAM1 (1:100; BD Biosciences, 553370; RRID: AB_394816), anti-ICAM1 (1:200; R&D Systems, AF796; RRID: AB_2248703), anti-ECAD (1:200; Cell Signaling Technology, 3195; RRID: AB_2291471), anti-LYVE1 (1:200; R&D Systems, AF2125; RRID: AB_2297188), anti-PROX1 (1:200; AngioBio, 11-002P; RRID: AB_10013720), anti-CD41 (1:200; Invitrogen, MA5-16875; RRID: AB_2538353), anti-VWF (1:200; Dako, A0082; RRID: AB_2315602), anti-CD68 (1:200; Bio-Rad, MCA1957; RRID: AB_322219), anti-CD45 (1:500; R&D Systems, AF114; RRID: AB_442146), anti-F4/80 (1:400; Cell Signaling Technology, 30325; RRID: AB_2798990), anti-MPO (1:200; Thermo Fisher Scientific, RB-373-A0; RRID: AB_59598), anti-TER119 (1:400; R&D Systems, MAB1125; RRID: AB_2297123) and anti-SMA (1:500; Sigma-Aldrich, A5228; RRID: AB_262054). Sections were washed three times with cold PBS and then incubated for 1 h at room temperature with secondary antibodies diluted to 1:500 in 1% BSA in PBS. Secondary antibodies used were: Cy3 donkey anti-rabbit IgG, Cy5 donkey anti-rabbit IgG, Alexa 488 donkey anti-goat IgG, Cy3 donkey anti-goat IgG, Cy5 donkey anti-goat IgG, Cy5 donkey anti-rat IgG (1:500 each; Jackson ImmunoResearch, 711-166-152, 711-175-152, 705-545-147, 705-166-147, 705-175-147, 712-175-153, respectively) and Alexa 488 donkey anti-rat IgG (1:500; Invitrogen, A21208). For nuclear counterstaining, sections were treated with 10 μg/ml Hoechst 33342 (Biotium, 40046) for 5 min. After immunostaining, slides were mounted using ProLong Gold antifade mountant (Thermo Fisher Scientific, P36930). Images were collected using either an Eclipse Ti-E epifluorescence microscope or an AX-R confocal microscope from Nikon and were analyzed with either Nikon Elements (v4) or Fiji software.

### LSEC isolation

LYVE1^+^ LSECs were isolated as previously described (Crosswhite et al., 2016). E12.5 embryonic livers were digested with digestion solution (1.5 mg/ml collagenase B, 1 U/ml DNase and 1.5 mg/ml dispase II in Dulbecco's Modified Eagle Medium) for 15 min at 37°C. After digestion, cells were pelleted by centrifugation at 300 ***g*** for 10 min at 4°C. Red blood cells were removed using 1 ml of ammonium-chloride-potassium lysing buffer (150 mmol/l ammonium chloride, 10 mmol/l sodium bicarbonate and 1 mmol/l EDTA; pH 7.4) for 5 min. The cell suspension was then strained through a 70-μm cell strainer and resuspended in a mixture of Dynabeads Protein G (Invitrogen, 10004D) and LYVE1 antibody (R&D Systems, AF2125) complexes, using 10 μl of beads and 1.3 μg of antibody per sample. The mixture was incubated for 30 min at 4°C. Immunoprecipitated LSECs and non-EC liver cells were separated with a magnetic stand and lysed in TRIzol (Invitrogen, 15596026) for gene expression analysis.

### Cell culture and siRNA transfection

The MS1 adult murine pancreatic EC line (ATCC, CRL-2279) was cultured in Dulbecco's Modified Eagle Medium supplemented with 5% fetal bovine serum. Cells were maintained in a 37°C incubator with a humidified atmosphere of 5% CO_2_ and tested to confirm freedom from *Mycoplasma* contamination. For siRNA-mediated gene knockdown, MS1 cells were plated in 12-well plates 1 day before being transfected with 50 pmol of BRG1*-*specific (Ambion, s73999), CHD4*-*specific (Ambion, s99014) or nonspecific control (Ambion, AM4635) siRNA oligos. Transfections were performed using Lipofectamine RNAiMAX Transfection Reagent (Invitrogen, 13778150), following the manufacturer's protocol. Twenty-four hours after transfection, cells were serum-starved in medium containing 0.5% fetal bovine serum. RNA was extracted for gene expression analysis 48 h post-transfection.

### RNA extraction and qRT-PCR

Total RNA was extracted from livers, LSECs or MS1 cells using TRIzol, followed by purification with the RNeasy Mini Prep Kit (QIAGEN, 74106). RNA samples (0.1-1 μg) were converted to cDNA using the iScript cDNA Synthesis Kit (Bio-Rad, 1708891). qRT-PCR analysis was performed using SsoAdvanced Universal SYBR Green Supermix (Bio-Rad, 1725274) and analyzed on a CFX96 Real-Time PCR thermocycler (Bio-Rad) according to the manufacturer's instructions. Target gene expression was normalized against at least two reference genes: *Actb*, *Gapdh*, *Rn18s* or *Rpl13a*. The primers used were as follows: *Brg1* forward (5′-CAGTGGCTCAAGGCTATCG-3′), *Brg1* reverse (5′-TGTCTCGCTTACGCTTACG-3′); *Chd4* forward (5′-TCCTCTGTCCACCATCATCA-3′), *Chd4* reverse (5′-ACCCAAGATGGCCATATCAA-3′); *Plaur* forward (5′-GGCTTAGATGTGCTGGGAAA-3′), *Plaur* reverse (5′-CAATGAGGCTGAGTTGAGCA-3′); *Stab2* forward (5′-ATTGCTCTGGCTGCCTACTC-3′), *Stab2* reverse (5′-GTTGGCTGGCTTCTCACATC-3′); *Afp* forward (5′-AGTTTCCAGAACCTGCCGAG-3′), *Afp* reverse (5′-ACCTTGTCGTACTGAGCAGC-3′); *Tnf* forward (5′-CAGGCGGTGCCTATGTCT-3′), *Tnf* reverse (5′-AGGGTCTGGGCCATAGAACT-3′); *Il6* forward (5′-CAAAGCCAGAGTCCTTCAGAG-3′), *Il6* reverse (5′-TGGTCCTTAGCCACTCCTTC-3′); *Il1b* forward (5′-ACCAACAAGTGATATTCTCCATG-3′), *Il1b* reverse (5′-ATCCACACTCTCCAGCTGC-3′); *Icam1* forward (5′-CAATTTCTCATGCCGCACAG-3′), *Icam1* reverse (5′-AGCTGGAAGATCGAAAGTCCG-3′); *Vcam1* forward (5′-TGAACCCAAACAGAGGCAGAGT-3′), *Vcam1* reverse (5′-GGTATCCCATCACTTGAGCAGG-3′); *Actb* forward (5′-TGTTACCAACTGGGACGACA-3′), *Actb* reverse (5′-GGGGTGTTGAAGGTCTCAAA-3′); *Gapdh* forward (5′-TCAACGGCACAGTCAAGG-3′), *Gapdh* reverse (5′-ACTCCACGACATACTCAGC-3′); *Rn18s* forward (5′-CCCGAAGCGTTTACTTTGAAA-3′), *Rn18s* reverse (5′-CGCGGTCCTATTCCATTATTC-3′); *Rpl13a* forward (5′-GGATCCCTCCACCCTATGACA-3′), *Rpl13a* reverse (5′-CTGGTACTTCCACCCGACCTC-3′). For all transcript analyses, transcript counts from mutant embryos were normalized to littermate controls to enable combined results across multiple litters. Statistical analysis was then performed after this normalization.

### ChIP-qPCR

ChIP experiments were performed using the Active Motif ChIP-IT^®^ Express Kit, following the manufacturer's instructions. MS1 cells were cultured in 150-mm dishes without reaching full confluency. Cells were prepared by fixing the monolayer with 1% formaldehyde in serum-free media for 10 min at room temperature. After washing with cold PBS, 1× glycine solution was applied to cells to inhibit cross-linking. Cells were then pelleted, resuspended in lysis buffer, and incubated on ice for 15 min. Nuclei were isolated through Dounce homogenization (ten strokes) and subsequently pelleted by centrifugation at 500 ***g*** at 4°C for 10 min. Pelleted nuclei were resuspended in 350 μl of shearing buffer and sonicated with a Misonix S-4000 sonicator (12 cycles of 20-second on/off pulses with an amplitude of 45) to achieve DNA fragments averaging 500 base pairs in length. Sheared chromatin was incubated with either 6 μg of IgG isotype control, anti-CHD4 (Abcam, ab70469; RRID: AB_2229454) or anti-BRG1 antibodies (Santa Cruz Biotechnology, sc-17796, RRID: AB_626762; Novus Biologicals, NB100-2594, RRID: AB_2191852) and rotated end-to-end at 4°C overnight. After incubation, precipitated samples were washed, eluted, and reverse cross-linked, with subsequent protein digestion performed using proteinase K. Interactions involving CHD4 and BRG1 were analyzed through qPCR. For the *Plaur* gene, the following primers were used: 5′-ACTGAGCCGCTCTGAGTGAT-3′ and 5′-CCAGGGGAAAAACAAGTTGA-3′. For the 215 bp region upstream of the *Icam1* gene (−*215-Icam1*), the following primers were used: 5′-ACTCACCTGCTGGTCTCTGA-3′ and 5′-GCCCCTGCGATCTAGGAATTT-3′. For the 72 bp region downstream of the *Icam1* gene (*+72-Icam1*), the following primers were used: 5′-CACGCTACCTCTGCTCCTG-3′ and 5′-TGGGTCCTGGAGTTTGGAGA-3′. For the exon 1 region of the *Tnf* gene, the following primers were used: 5′-GCAGGTTCTGTCCCTTTCAC-3′ and 5′-AGTGCCTCTTCTGCCAGTTC-3′. For the exon 4 region of the *Tnf* gene, the following primers were used: 5′-TATGGCTCAGGGTCCAACTC-3′ and 5′-GCTCCAGTGAATTCGGAAAG-3′.

### LSEC RNA-Seq and analysis

LSECs were isolated from E12.5 embryonic livers as described in the LSEC isolation section, with a few modifications. Transcription inhibitors, including 5 μg/ml actinomycin D, 10 μM triptolide and 10 μg/ml anisomycin, were added to the digestion solution (Ocañas et al., 2022). Additionally, CD146 MicroBeads (Miltenyi, 130-092-007) were utilized to separate LSECs from non-EC liver cells. Total RNA was extracted following previously established methods, which included an on-column DNase digestion step. RNA quality was assessed using RNA ScreenTape on a TapeStation 4200 (Agilent). Library preparation and RNA sequencing (Novaseq PE150, 20 million reads) were conducted by the Clinical Genomics Center at the Oklahoma Medical Research Foundation. Repeated guanines in raw FASTQ reads from all samples were uniformly trimmed, and quality control measures were performed. Alignment to the mouse genome (GRCm39/mm39) and differential expression analysis were executed using CLC Genomics Workbench (QIAGEN). Genes exhibiting a log_2_ fold change >1 and an adjusted *P*-value <0.05 compared to LSEC-Control were classified as DEGs. Volcano plots, principal component analysis, and heatmaps were generated using R. GSEA was performed by computing overlaps with LSEC datasets and the Molecular Signatures Database gene sets using the GSEA software tool from the Broad Institute (Subramanian et al., 2005). GO and Kyoto Encyclopedia of Genes and Genomes (KEGG) pathway enrichment analyses were performed using the web-based portal Metascape (Zhou et al., 2019).

### Statistics

Prism 10.0 software (GraphPad) was used for all statistical assessments. For *in vivo* experiments, each data point represents a biological replicate from a single embryo. For *in vitro* experiments using cultured MS1 cells, each data point represents a technical replicate from a single lot of cells. The ‘*n*’ numbers in the figure legends reflect these distinctions. Statistically analyzed data are presented as mean (±s.d.), and associated numbers indicate adjusted *P*-values. Data points identified as outliers by the ROUT method in Prism were excluded. Tests for normality (D'Agostino–Pearson and Shapiro–Wilk) and equal variance (Brown–Forsythe) were used to determine the appropriate parametric or non-parametric statistical model. In general, the comparison between two groups was assessed by one-sample *t*-tests (Figs 2B and 5A). Comparison of multiple means was made using a one-way ANOVA or a Kruskal–Wallis test, as appropriate, with relevant post-hoc tests (Dunnett's or Dunn's, respectively).

### Use of artificial intelligence tools

This article was written with the assistance of ChatGPT and Grammarly, which were used to correct grammatical errors and enhance clarity. The authors subsequently reviewed and edited the content as necessary and take full responsibility for the publication's final content.

## Supplementary Material



10.1242/develop.205538_sup1 Supplementary information

## References

[DEV205538C1] Alva, J. A., Zovein, A. C., Monvoisin, A., Murphy, T., Salazar, A., Harvey, N. L., Carmeliet, P. and Iruela-Arispe, M. L. (2006). VE-cadherin-Cre-recombinase transgenic mouse: a tool for lineage analysis and gene deletion in endothelial cells. *Dev. Dyn.* 235, 759-767. 10.1002/dvdy.20643 16450386

[DEV205538C2] Amenta, P. S. and Harrison, D. (1997). Expression and potential role of the extracellular matrix in hepatic ontogenesis: a review. *Microsc. Res. Tech.* 39, 372-386. 10.1002/(SICI)1097-0029(19971115)39:4<372::AID-JEMT7>3.0.CO;2-J 9407547

[DEV205538C3] Augustin, H. G. and Koh, G. Y. (2017). Organotypic vasculature: from descriptive heterogeneity to functional pathophysiology. *Science* 357, eaal2379. 10.1126/science.aal2379 28775214

[DEV205538C4] Beg, A. A., Sha, W. C., Bronson, R. T., Ghosh, S. and Baltimore, D. (1995). Embryonic lethality and liver degeneration in mice lacking the RelA component of NF-κB. *Nature* 376, 167-170. 10.1038/376167a0 7603567

[DEV205538C5] Bishop, A. J., Israels, L. G., Chernick, V. and Israels, E. D. (1971). Placental transfer of intravascular coagulation between mother and fetus. *Pediatr. Res.* 5, 113-125. 10.1203/00006450-197103000-00003

[DEV205538C6] Blasi, F. and Carmeliet, P. (2002). uPAR: a versatile signalling orchestrator. *Nat. Rev. Mol. Cell Biol.* 3, 932-943. 10.1038/nrm977 12461559

[DEV205538C7] Boulter, L., Lu, W.-Y. and Forbes, S. J. (2013). Differentiation of progenitors in the liver: a matter of local choice. *J. Clin. Invest.* 123, 1867-1873. 10.1172/JCI66026 23635784 PMC3635730

[DEV205538C8] Brenner, D., Blaser, H. and Mak, T. W. (2015). Regulation of tumour necrosis factor signalling: live or let die. *Nat. Rev. Immunol.* 15, 362-374. 10.1038/nri3834 26008591

[DEV205538C9] Bui, T. M., Wiesolek, H. L. and Sumagin, R. (2020). ICAM-1: a master regulator of cellular responses in inflammation, injury resolution, and tumorigenesis. *J. Leukoc. Biol.* 108, 787-799. 10.1002/JLB.2MR0220-549R 32182390 PMC7977775

[DEV205538C10] Burysek, L., Syrovets, T. and Simmet, T. (2002). The serine protease plasmin triggers expression of MCP-1 and CD40 in human primary monocytes via activation of p38 MAPK and janus kinase (JAK)/STAT signaling pathways. *J. Biol. Chem.* 277, 33509-33517. 10.1074/jbc.M201941200 12093796

[DEV205538C11] Chang, W. C., Shi, G. Y., Chow, Y. H., Chang, L. C., Hau, J. S., Lin, M. T., Jen, C. J., Wing, L. Y. and Wu, H. L. (1993). Human plasmin induces a receptor-mediated arachidonate release coupled with G proteins in endothelial cells. *Am. J. Physiol.* 264, C271-C281. 10.1152/ajpcell.1993.264.2.C271 8383426

[DEV205538C12] Chang, S., Young, B. D., Li, S., Qi, X., Richardson, J. A. and Olson, E. N. (2006). Histone deacetylase 7 maintains vascular integrity by repressing matrix metalloproteinase 10. *Cell* 126, 321-334. 10.1016/j.cell.2006.05.040 16873063

[DEV205538C13] Clark, P. R., Manes, T. D., Pober, J. S. and Kluger, M. S. (2007). Increased ICAM-1 expression causes endothelial cell leakiness, cytoskeletal reorganization and junctional alterations. *J. Invest. Dermatol.* 127, 762-774. 10.1038/sj.jid.5700670 17195014

[DEV205538C14] Colijn, S., Gao, S., Ingram, K. G., Menendez, M., Muthukumar, V., Silasi-Mansat, R., Chmielewska, J. J., Hinsdale, M., Lupu, F. and Griffin, C. T. (2020). The NuRD chromatin-remodeling complex enzyme CHD4 prevents hypoxia-induced endothelial *Ripk3* transcription and murine embryonic vascular rupture. *Cell Death Differ.* 27, 618-631. 10.1038/s41418-019-0376-8 31235857 PMC7206092

[DEV205538C15] Collins, A., Mitchell, C. A. and Passegue, E. (2021). Inflammatory signaling regulates hematopoietic stem and progenitor cell development and homeostasis. *J. Exp. Med.* 218, e20201545. 10.1084/jem.20201545 34129018 PMC8210624

[DEV205538C16] Crosswhite, P. L., Podsiadlowska, J. J., Curtis, C. D., Gao, S., Xia, L., Srinivasan, R. S. and Griffin, C. T. (2016). CHD4-regulated plasmin activation impacts lymphovenous hemostasis and hepatic vascular integrity. *J. Clin. Invest.* 126, 2254-2266. 10.1172/JCI84652 27140400 PMC4887170

[DEV205538C17] Curtis, C. D. and Griffin, C. T. (2012). The chromatin-remodeling enzymes BRG1 and CHD4 antagonistically regulate vascular Wnt signaling. *Mol. Cell. Biol.* 32, 1312-1320. 10.1128/MCB.06222-11 22290435 PMC3302445

[DEV205538C18] Curtis, C. D., Davis, R. B., Ingram, K. G. and Griffin, C. T. (2012). Chromatin-remodeling complex specificity and embryonic vascular development. *Cell. Mol. Life Sci.* 69, 3921-3931. 10.1007/s00018-012-1023-4 22618247 PMC3661716

[DEV205538C19] Davis, G. E. and Senger, D. R. (2005). Endothelial extracellular matrix: biosynthesis, remodeling, and functions during vascular morphogenesis and neovessel stabilization. *Circ. Res.* 97, 1093-1107. 10.1161/01.RES.0000191547.64391.e3 16306453

[DEV205538C20] Davis, R. B., Curtis, C. D. and Griffin, C. T. (2013). BRG1 promotes COUP-TFII expression and venous specification during embryonic vascular development. *Development* 140, 1272-1281. 10.1242/dev.087379 23406903 PMC3585661

[DEV205538C21] De Boer, J., Williams, A., Skavdis, G., Harker, N., Coles, M., Tolaini, M., Norton, T., Williams, K., Roderick, K., Potocnik, A. J. et al. (2003). Transgenic mice with hematopoietic and lymphoid specific expression of Cre. *Eur. J. Immunol.* 33, 314-325. 10.1002/immu.200310005 12548562

[DEV205538C22] Delgado-Olguín, P., Dang, L. T., He, D., Thomas, S., Chi, L., Sukonnik, T., Khyzha, N., Dobenecker, M.-W., Fish, J. E. and Bruneau, B. G. (2014). Ezh2-mediated repression of a transcriptional pathway upstream of *Mmp9* maintains integrity of the developing vasculature. *Development* 141, 4610-4617. 10.1242/dev.112607 25359725 PMC4302930

[DEV205538C23] Doeuvre, L., Plawinski, L., Goux, D., Vivien, D. and Anglés-Cano, E. (2010). Plasmin on adherent cells: from microvesiculation to apoptosis. *Biochem. J.* 432, 365-373. 10.1042/BJ20100561 20846121 PMC3124466

[DEV205538C24] Doi, T. S., Marino, M. W., Takahashi, T., Yoshida, T., Sakakura, T., Old, L. J. and Obata, Y. (1999). Absence of tumor necrosis factor rescues RelA-deficient mice from embryonic lethality. *Proc. Natl. Acad. Sci. USA* 96, 2994-2999. 10.1073/pnas.96.6.2994 10077625 PMC15883

[DEV205538C25] Dragoni, S., Hudson, N., Kenny, B.-A., Burgoyne, T., McKenzie, J. A., Gill, Y., Blaber, R., Futter, C. E., Adamson, P., Greenwood, J. et al. (2017). Endothelial MAPKs direct ICAM-1 signaling to divergent inflammatory functions. *J. Immunol.* 198, 4074-4085. 10.4049/jimmunol.1600823 28373581 PMC5421301

[DEV205538C26] Draxler, D. F., Sashindranath, M. and Medcalf, R. L. (2017). Plasmin: a modulator of immune function. *Semin. Thromb. Hemost.* 43, 143-153. 10.1055/s-0036-1586227 27677178

[DEV205538C27] Espin-Palazon, R., Stachura, D. L., Campbell, C. A., García-Moreno, D., Del Cid, N., Kim, A. D., Candel, S., Meseguer, J., Mulero, V. and Traver, D. (2014). Proinflammatory signaling regulates hematopoietic stem cell emergence. *Cell* 159, 1070-1085. 10.1016/j.cell.2014.10.031 25416946 PMC4243083

[DEV205538C28] Fang, F., Chen, D., Yu, L., Dai, X., Yang, Y., Tian, W., Cheng, X., Xu, H., Weng, X., Fang, M. et al. (2013). Proinflammatory stimuli engage Brahma related gene 1 and Brahma in endothelial injury. *Circ. Res.* 113, 986-996. 10.1161/CIRCRESAHA.113.301296 23963727 PMC4049295

[DEV205538C29] Gao, S., Silasi-Mansat, R., Behar, A. R., Lupu, F. and Griffin, C. T. (2018). Excessive plasmin compromises hepatic sinusoidal vascular integrity after acetaminophen overdose. *Hepatology* 68, 1991-2003. 10.1002/hep.30070 29729197 PMC6204085

[DEV205538C30] Gao, S., Shi, Q., Zhang, Y., Liang, G., Kang, Z., Huang, B., Ma, D., Wang, L., Jiao, J., Fang, X. et al. (2022). Identification of HSC/MPP expansion units in fetal liver by single-cell spatiotemporal transcriptomics. *Cell Res.* 32, 38-53. 10.1038/s41422-021-00540-7 34341490 PMC8724330

[DEV205538C31] Georgiades, P., Ogilvy, S., Duval, H., Licence, D. R., Charnock-Jones, D. S., Smith, S. K. and Print, C. G. (2002). *VavCre* transgenic mice: a tool for mutagenesis in hematopoietic and endothelial lineages. *Genesis* 34, 251-256. 10.1002/gene.10161 12434335

[DEV205538C32] Goddi, A., Schroedl, L., Brey, E. M. and Cohen, R. N. (2021). Laminins in metabolic tissues. *Metabolism* 120, 154775. 10.1016/j.metabol.2021.154775 33857525

[DEV205538C33] Griffin, C. T., Brennan, J. and Magnuson, T. (2008). The chromatin-remodeling enzyme BRG1 plays an essential role in primitive erythropoiesis and vascular development. *Development* 135, 493-500. 10.1242/dev.010090 18094026 PMC2459551

[DEV205538C34] Griffin, C. T., Curtis, C. D., Davis, R. B., Muthukumar, V. and Magnuson, T. (2011). The chromatin-remodeling enzyme BRG1 modulates vascular Wnt signaling at two levels. *Proc. Natl. Acad. Sci. USA* 108, 2282-2287. 10.1073/pnas.1013751108 21262838 PMC3038709

[DEV205538C35] Guerra-Espinosa, C., Jiménez-Fernández, M., Sánchez-Madrid, F. and Serrador, J. M. (2024). ICAMs in immunity, intercellular adhesion and communication. *Cells* 13, 339. 10.3390/cells13040339 38391953 PMC10886500

[DEV205538C36] Gupta, P., Oegema, T. R., Jr, Brazil, J. J., Dudek, A. Z., Slungaard, A. and Verfaillie, C. M. (1998). Structurally specific heparan sulfates support primitive human hematopoiesis by formation of a multimolecular stem cell niche. *Blood* 92, 4641-4651. 10.1182/blood.V92.12.4641 9845530

[DEV205538C37] Gyetko, M. R., Sud, S., Kendall, T., Fuller, J. A., Newstead, M. W. and Standiford, T. J. (2000). Urokinase receptor-deficient mice have impaired neutrophil recruitment in response to pulmonary Pseudomonas aeruginosa infection. *J. Immunol.* 165, 1513-1519. 10.4049/jimmunol.165.3.1513 10903758

[DEV205538C38] Gyetko, M. R., Sud, S., Sonstein, J., Polak, T., Sud, A. and Curtis, J. L. (2001). Antigen-driven lymphocyte recruitment to the lung is diminished in the absence of urokinase-type plasminogen activator (uPA) receptor, but is independent of uPA. *J. Immunol.* 167, 5539-5542. 10.4049/jimmunol.167.10.5539 11698423

[DEV205538C39] Hamada, M., Varkoly, K. S., Riyadh, O., Beladi, R., Munuswamy-Ramanujam, G., Rawls, A., Wilson-Rawls, J., Chen, H., McFadden, G. and Lucas, A. R. (2024). Urokinase-Type Plasminogen Activator Receptor (uPAR) in inflammation and disease: a unique inflammatory pathway activator. *Biomedicines* 12, 1167. 10.3390/biomedicines12061167 38927374 PMC11201033

[DEV205538C40] Ingram, K. G., Curtis, C. D., Silasi-Mansat, R., Lupu, F. and Griffin, C. T. (2013). The NuRD chromatin-remodeling enzyme CHD4 promotes embryonic vascular integrity by transcriptionally regulating extracellular matrix proteolysis. *PLoS Genet.* 9, e1004031. 10.1371/journal.pgen.1004031 24348274 PMC3861115

[DEV205538C41] Kamiya, A. and Gonzalez, F. J. (2004). TNF-α regulates mouse fetal hepatic maturation induced by oncostatin M and extracellular matrices. *Hepatology* 40, 527-536. 10.1002/hep.20362 15349890

[DEV205538C42] Kesanakurti, D., Chetty, C., Rajasekhar Maddirela, D., Gujrati, M. and Rao, J. S. (2013). Essential role of cooperative NF-κB and Stat3 recruitment to ICAM-1 intronic consensus elements in the regulation of radiation-induced invasion and migration in glioma. *Oncogene* 32, 5144-5155. 10.1038/onc.2012.546 23178493 PMC3664652

[DEV205538C43] Kourouklis, A. P., Kaylan, K. B. and Underhill, G. H. (2016). Substrate stiffness and matrix composition coordinately control the differentiation of liver progenitor cells. *Biomaterials* 99, 82-94. 10.1016/j.biomaterials.2016.05.016 27235994

[DEV205538C44] Li, Q., van Antwerp, D., Mercurio, F., Lee, K.-F. and Verma, I. M. (1999). Severe liver degeneration in mice lacking the IκB kinase 2 gene. *Science* 284, 321-325. 10.1126/science.284.5412.321 10195897

[DEV205538C45] Li, Q., Syrovets, T., Simmet, T., Ding, J., Xu, J., Chen, W., Zhu, D. and Gao, P. (2013). Plasmin induces intercellular adhesion molecule 1 expression in human endothelial cells via nuclear factor-κB/mitogen-activated protein kinases-dependent pathways. *Exp. Biol. Med.* 238, 176-186. 10.1177/1535370212473700 23576799

[DEV205538C46] Li, Y., Esain, V., Teng, L., Xu, J., Kwan, W., Frost, I. M., Yzaguirre, A. D., Cai, X., Cortes, M., Maijenburg, M. W. et al. (2014). Inflammatory signaling regulates embryonic hematopoietic stem and progenitor cell production. *Genes Dev.* 28, 2597-2612. 10.1101/gad.253302.114 25395663 PMC4248291

[DEV205538C47] Liu, Y.-F., Zhang, S.-Y., Chen, Y.-Y., Shi, K., Zou, B., Liu, J., Yang, Q., Jiang, H., Wei, L., Li, C.-Z. et al. (2018). ICAM-1 deficiency in the bone marrow niche impairs quiescence and repopulation of hematopoietic stem cells. *Stem Cell Rep.* 11, 258-273. 10.1016/j.stemcr.2018.05.016 PMC6117479 29937143

[DEV205538C48] Löhler, J., Timpl, R. and Jaenisch, R. (1984). Embryonic lethal mutation in mouse collagen I gene causes rupture of blood vessels and is associated with erythropoietic and mesenchymal cell death. *Cell* 38, 597-607. 10.1016/0092-8674(84)90514-2 6467375

[DEV205538C49] Lorenz, L., Axnick, J., Buschmann, T., Henning, C., Urner, S., Fang, S., Nurmi, H., Eichhorst, N., Holtmeier, R., Bódis, K. et al. (2018). Mechanosensing by β1 integrin induces angiocrine signals for liver growth and survival. *Nature* 562, 128-132. 10.1038/s41586-018-0522-3 30258227

[DEV205538C50] Lorenzini, S., Bird, T. G., Boulter, L., Bellamy, C., Samuel, K., Aucott, R., Clayton, E., Andreone, P., Bernardi, M., Golding, M. et al. (2010). Characterisation of a stereotypical cellular and extracellular adult liver progenitor cell niche in rodents and diseased human liver. *Gut* 59, 645-654. 10.1136/gut.2009.182345 20427399 PMC3034133

[DEV205538C51] Mahony, C. B., Cacialli, P., Pasche, C., Monteiro, R., Savvides, S. N. and Bertrand, J. Y. (2021). *Hapln1b*, a central organizer of the ECM, modulates kit signaling to control developmental hematopoiesis in zebrafish. *Blood Adv.* 5, 4935-4948. 10.1182/bloodadvances.2020001524 34543380 PMC9152995

[DEV205538C52] Menendez, M. T., Ong, E.-C., Shepherd, B. T., Muthukumar, V., Silasi-Mansat, R., Lupu, F. and Griffin, C. T. (2017). BRG1 (Brahma-Related Gene 1) promotes endothelial *Mrtf* transcription to establish embryonic capillary integrity. *Arterioscler. Thromb. Vasc. Biol.* 37, 1674-1682. 10.1161/ATVBAHA.117.309785 28729363 PMC5570645

[DEV205538C53] Mesquita Peixoto, M., Soares-Da-Silva, F., Bonnet, V., Zhou, Y., Ronteix, G., Santos, R. F., Mailhe, M. P., Nogueira, G., Feng, X., Pereira, J. P. et al. (2025). Spatiotemporal dynamics of fetal liver hematopoietic niches. *J. Exp. Med.* 222, e20240592. 10.1084/jem.20240592 39775824 PMC11706214

[DEV205538C54] Morris, S. A., Baek, S., Sung, M.-H., John, S., Wiench, M., Johnson, T. A., Schiltz, R. L. and Hager, G. L. (2014). Overlapping chromatin-remodeling systems collaborate genome wide at dynamic chromatin transitions. *Nat. Struct. Mol. Biol.* 21, 73-81. 10.1038/nsmb.2718 24317492 PMC3947387

[DEV205538C55] Naito, M., Zager, R. A. and Bomsztyk, K. (2009). BRG1 increases transcription of proinflammatory genes in renal ischemia. *J. Am. Soc. Nephrol.* 20, 1787-1796. 10.1681/ASN.2009010118 19556365 PMC2723991

[DEV205538C56] Ocañas, S. R., Pham, K. D., Blankenship, H. E., Machalinski, A. H., Chucair-Elliott, A. J. and Freeman, W. M. (2022). Minimizing the *ex vivo* confounds of cell-isolation techniques on transcriptomic and translatomic profiles of purified microglia. *eNeuro* 9, ENEURO.0348-21.2022. 10.1523/ENEURO.0348-21.2022 PMC8970438 35228310

[DEV205538C57] Orelio, C., Peeters, M., Haak, E., Van Der Horn, K. and Dzierzak, E. (2009). Interleukin-1 regulates hematopoietic progenitor and stem cells in the midgestation mouse fetal liver. *Haematologica* 94, 462-469. 10.3324/haematol.13728 19229053 PMC2663609

[DEV205538C58] Ploplis, V. A., Carmeliet, P., Vazirzadeh, S., Van Vlaenderen, I., Moons, L., Plow, E. F. and Collen, D. (1995). Effects of disruption of the plasminogen gene on thrombosis, growth, and health in mice. *Circulation* 92, 2585-2593. 10.1161/01.CIR.92.9.2585 7586361

[DEV205538C59] Pöschl, E., Schlötzer-Schrehardt, U., Brachvogel, B., Saito, K., Ninomiya, Y. and Mayer, U. (2004). Collagen IV is essential for basement membrane stability but dispensable for initiation of its assembly during early development. *Development* 131, 1619-1628. 10.1242/dev.01037 14998921

[DEV205538C60] Ramirez-Carrozzi, V. R., Nazarian, A. A., Li, C. C., Gore, S. L., Sridharan, R., Imbalzano, A. N. and Smale, S. T. (2006). Selective and antagonistic functions of SWI/SNF and Mi-2β nucleosome remodeling complexes during an inflammatory response. *Genes Dev.* 20, 282-296. 10.1101/gad.1383206 16452502 PMC1361700

[DEV205538C61] Rezvani, M., Quach, S., Lewis, K., Saiki, N., Xue, C., Kimura, M., Iwasawa, K., Weihs, J., Elzobair, T., Al Reza, H. et al. (2026). Modeling immune lineage co-development in human pluripotent stem cell-derived liver organoids. *J. Hepatol.* 84, 946-961. 10.1016/j.jhep.2025.11.018 41418844 PMC13033979

[DEV205538C62] Rosenfeld, M. E., Prichard, L., Shiojiri, N. and Fausto, N. (2000). Prevention of hepatic apoptosis and embryonic lethality in *RelA/TNFR-*1 double knockout mice. *Am. J. Pathol.* 156, 997-1007. 10.1016/S0002-9440(10)64967-X 10702415 PMC1876833

[DEV205538C63] Rossignol, P., Anglès-Cano, E. and Lijnen, H. R. (2006). Plasminogen activator inhibitor-1 impairs plasminogen activation-mediated vascular smooth muscle cell apoptosis. *Thromb. Haemost.* 96, 665-670. 10.1160/TH06-06-0321 17080225 PMC2237888

[DEV205538C64] Rudolph, D., Yeh, W.-C., Wakeham, A., Rudolph, B., Nallainathan, D., Potter, J., Elia, A. J. and Mak, T. W. (2000). Severe liver degeneration and lack of NF-κB activation in NEMO/IKKγ-deficient mice. *Genes Dev.* 14, 854-862. 10.1101/gad.14.7.854 10766741 PMC316493

[DEV205538C65] Sano, K., Nagaki, M., Sugiyama, A., Hatakeyama, H., Ohnishi, H., Muto, Y. and Moriwaki, H. (1999). Effects of cytokines on the binding of leukocytes to cultured rat hepatocytes and on the expression of ICAM-1 by hepatocytes. *Dig. Dis. Sci.* 44, 796-805. 10.1023/A:1026682329876 10219841

[DEV205538C66] Satoh, S., Nussler, A. K., Liu, Z. Z. and Thomson, A. W. (1994). Proinflammatory cytokines and endotoxin stimulate ICAM-1 gene expression and secretion by normal human hepatocytes. *Immunology* 82, 571-576. 7835919 PMC1414914

[DEV205538C67] Shi, W., Scialdone, A. P., Emerson, J. I., Mei, L., Wasson, L. K., Davies, H. A., Seidman, C. E., Seidman, J. G., Cook, J. G. and Conlon, F. L. (2023). Missense mutation in human CHD4 causes ventricular noncompaction by repressing ADAMTS1. *Circ. Res.* 133, 48-67. 10.1161/CIRCRESAHA.122.322223 37254794 PMC10284140

[DEV205538C68] Si-Tayeb, K., Lemaigre, F. P. and Duncan, S. A. (2010). Organogenesis and development of the liver. *Dev. Cell* 18, 175-189. 10.1016/j.devcel.2010.01.011 20159590

[DEV205538C69] Smith, H. W. and Marshall, C. J. (2010). Regulation of cell signalling by uPAR. *Nat. Rev. Mol. Cell Biol.* 11, 23-36. 10.1038/nrm2821 20027185

[DEV205538C70] Soares-Da-Silva, F., Peixoto, M., Cumano, A. and Pinto-do-Ó, P. (2020). Crosstalk between the hepatic and hematopoietic systems during embryonic development. *Front. Cell. Dev. Biol.* 8, 612. 10.3389/fcell.2020.00612 32793589 PMC7387668

[DEV205538C71] Subramanian, A., Tamayo, P., Mootha, V. K., Mukherjee, S., Ebert, B. L., Gillette, M. A., Paulovich, A., Pomeroy, S. L., Golub, T. R., Lander, E. S. et al. (2005). Gene set enrichment analysis: a knowledge-based approach for interpreting genome-wide expression profiles. *Proc. Natl. Acad. Sci. USA* 102, 15545-15550. 10.1073/pnas.0506580102 16199517 PMC1239896

[DEV205538C72] Sumagin, R., Lomakina, E. and Sarelius, I. H. (2008). Leukocyte-endothelial cell interactions are linked to vascular permeability via ICAM-1-mediated signaling. *Am. J. Physiol Heart Circ. Physiol.* 295, H969-H977. 10.1152/ajpheart.00400.2008 18641276 PMC2544502

[DEV205538C73] Sumi-Ichinose, C., Ichinose, H., Metzger, D. and Chambon, P. (1997). SNF2β-BRG1 is essential for the viability of F9 murine embryonal carcinoma cells. *Mol. Cell. Biol.* 17, 5976-5986. 10.1128/MCB.17.10.5976 9315656 PMC232446

[DEV205538C74] Syrovets, T., Jendrach, M., Rohwedder, A., Schüle, A. and Simmet, T. (2001). Plasmin-induced expression of cytokines and tissue factor in human monocytes involves AP-1 and IKKβ-mediated NF-κB activation. *Blood* 97, 3941-3950. 10.1182/blood.V97.12.3941 11389038

[DEV205538C75] Syrovets, T., Lunov, O. and Simmet, T. (2012). Plasmin as a proinflammatory cell activator. *J. Leukoc. Biol.* 92, 509-519. 10.1189/jlb.0212056 22561604

[DEV205538C76] Tanimizu, N., Kikkawa, Y., Mitaka, T. and Miyajima, A. (2012). α1- and α5-containing laminins regulate the development of bile ducts via β1 integrin signals. *J. Biol. Chem.* 287, 28586-28597. 10.1074/jbc.M112.350488 22761447 PMC3436529

[DEV205538C77] Tiegs, G. and Horst, A. K. (2022). TNF in the liver: targeting a central player in inflammation. *Semin. Immunopathol.* 44, 445-459. 10.1007/s00281-022-00910-2 35122118 PMC9256556

[DEV205538C78] Wang, B., Kaufmann, B., Mogler, C., Zhong, S., Yin, Y., Cheng, Z., Schmid, R. M., Friess, H., Huser, N., von Figura, G. et al. (2023). Hepatocellular Brg1 promotes CCl4-induced liver inflammation, ECM accumulation and fibrosis in mice. *PLoS ONE* 18, e0294257. 10.1371/journal.pone.0294257 38033027 PMC10688683

[DEV205538C79] Wiley, M. M., Muthukumar, V., Griffin, T. M. and Griffin, C. T. (2015). SWI/SNF chromatin-remodeling enzymes Brahma-Related Gene 1 (BRG1) and Brahma (BRM) are dispensable in multiple models of postnatal angiogenesis but are required for vascular integrity in infant mice. *J. Am. Heart. Assoc.* 4, e001972. 10.1161/JAHA.115.001972 25904594 PMC4579958

[DEV205538C80] Williams, C. J., Naito, T., Arco, P. G.-D., Seavitt, J. R., Cashman, S. M., De Souza, B., Qi, X., Keables, P., Von Andrian, U. H. and Georgopoulos, K. (2004). The chromatin remodeler Mi-2β is required for CD4 expression and T cell development. *Immunity* 20, 719-733. 10.1016/j.immuni.2004.05.005 15189737

[DEV205538C81] Wójciak-Stothard, B., Entwistle, A., Garg, R. and Ridley, A. J. (1998). Regulation of TNF-alpha-induced reorganization of the actin cytoskeleton and cell-cell junctions by Rho, Rac, and Cdc42 in human endothelial cells. *J. Cell. Physiol.* 176, 150-165. 10.1002/(SICI)1097-4652(199807)176:1<150::AID-JCP17>3.0.CO;2-B 9618155

[DEV205538C82] Wu, M.-L., Wheeler, K., Silasi, R., Lupu, F. and Griffin, C. T. (2024). Endothelial chromatin-remodeling enzymes regulate the production of critical ECM components during murine lung development. *Arterioscler. Thromb. Vasc. Biol.* 44, 1784-1798. 10.1161/ATVBAHA.124.320881 38868942 PMC11624602

[DEV205538C83] You, K., Gu, H., Yuan, Z. and Xu, X. (2021). Tumor necrosis factor alpha signaling and organogenesis. *Front. Cell Dev. Biol.* 9, 727075. 10.3389/fcell.2021.727075 34395451 PMC8361451

[DEV205538C84] Zhou, Y., Zhou, B., Pache, L., Chang, M., Khodabakhshi, A. H., Tanaseichuk, O., Benner, C. and Chanda, S. K. (2019). Metascape provides a biologist-oriented resource for the analysis of systems-level datasets. *Nat. Commun.* 10, 1523. 10.1038/s41467-019-09234-6 30944313 PMC6447622

