# Supplementary material for: Plasmin and sterile inflammation jointly drive fatal embryonic liver degeneration in endothelial chromatin remodeler mutant mice
Source: Development. 2026 May 28;153(16):dev205538. doi: 10.1242/dev.205538 (PMC13286354; doi:10.1242/dev.205538)
Supplement: Supplementary information [file develop-153-205538-s1.pdf]

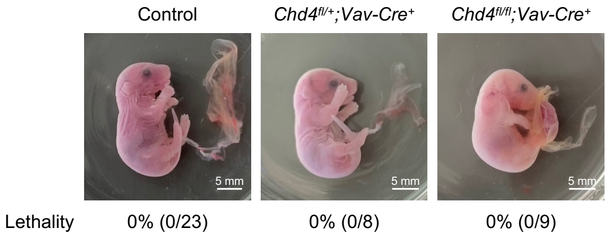

Fig. S1. Appearance and lethality rate of *Chd4$^{fl/fl}$;Vav-Cre^{+}* embryos. Representative images and lethality rates for littermate control and *Chd4$^{fl/fl}$;Vav-Cre^{+}* embryos at embryonic day (E) 18.5. N≥8 embryos for each genotype.

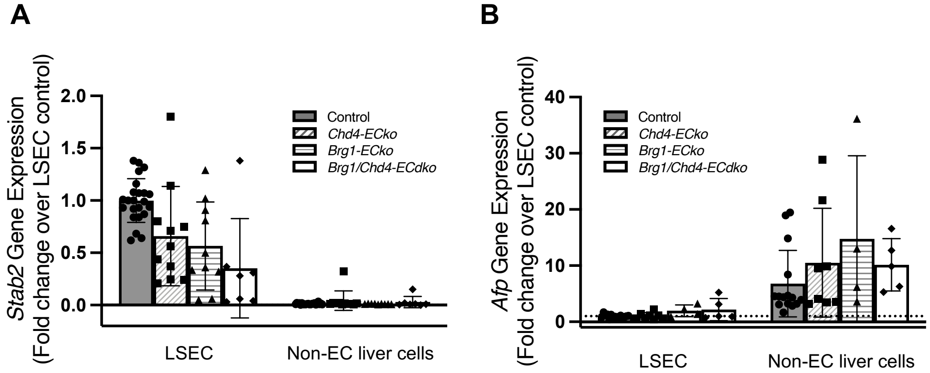

Fig. S2. Gene transcripts of liver sinusoidal endothelial cells (LSECs) and non-EC liver cells at E12.5. LSEC marker gene *Stab2* **(A)** and hepatocyte marker gene *Afp* **(B)** levels in LSECs and non-EC liver cells from control and mutant livers. N≥4 embryos, shown as individual symbols. Data are represented as mean (±s.d.).

Development • Supplementary information

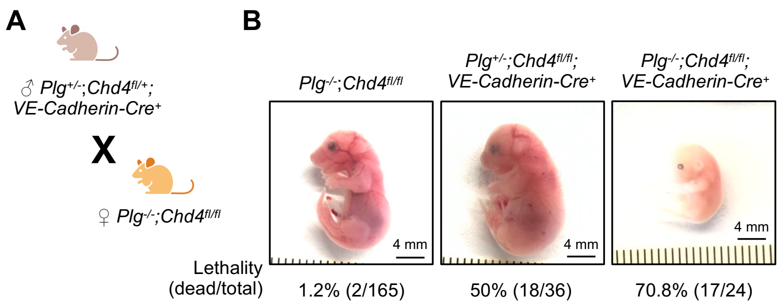

Fig. S3. Lethality of different doses of plasminogen-deficient *Chd4* mutant embryos generated from plasminogen null dams. A. A schematic representation of the crosses used to generate plasminogen (*Plg*)-deficient *Chd4*$^{fl/fl}$*;VE-cadherin-Cre*$^{+}$ embryos from *Plg* null dams is shown. **B.** Representative images and the lethality rate of control, *Plg*$^{+/-}$*; Chd4*$^{fl/fl}$*;VE-cadherin-Cre*$^{+}$, and *Plg*$^{-/-}$*;Chd4*$^{fl/fl}$*;VE-cadherin-Cre*$^{+}$ embryos at E17.5.

Development • Supplementary information

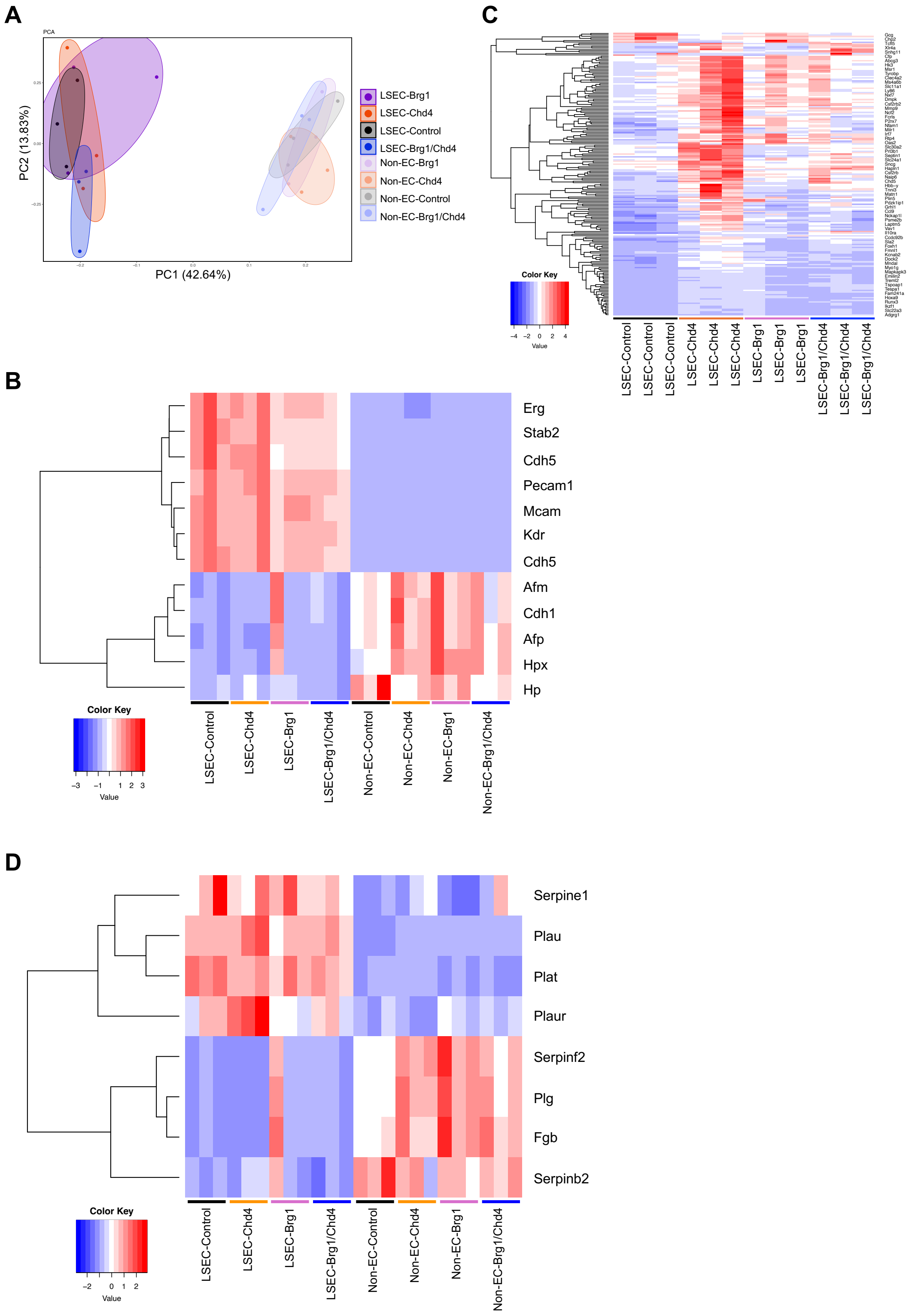

Development • Supplementary information

Fig. S4. Transcription profiles from RNA-sequencing of LSEC and non-EC samples from control and mutant livers at E12.5. A. Principal component analysis (PCA) of RNAsequencing samples from LSEC and non-EC groups, with outputs from 18,241 genes. **B.** Heatmap illustrating gene expression of EC and hepatic cell markers in LSEC and non-EC samples. **C.** Heatmap showing differentially expressed genes (DEG) in LSECs between control and *Chd4-ECko* LSECs. DEG were defined as having more than a twofold change in expression in *Chd4-ECko* LSECs relative to control LSECs, with an adjusted p-value <0.05. **D.** Heatmap showing gene expression of plasmin activation pathway molecules in LSEC and non-EC samples.

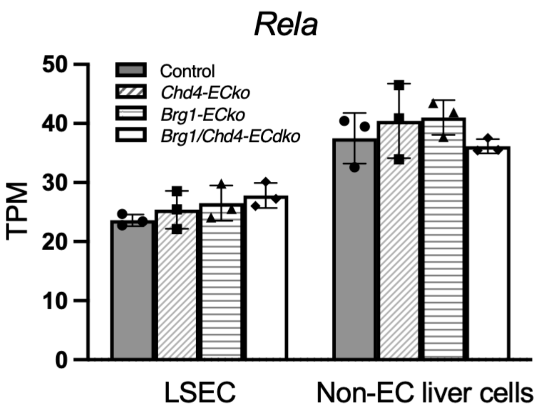

Fig. S5. *Rela* transcripts in LSECs and non-EC liver cells at E12.5. *Rela* transcripts gleaned from RNA-sequencing data. TPM: transcripts per million mapped reads. Each symbol represents data from a separate embryo. Data are represented as mean (±s.d.).

Development • Supplementary information

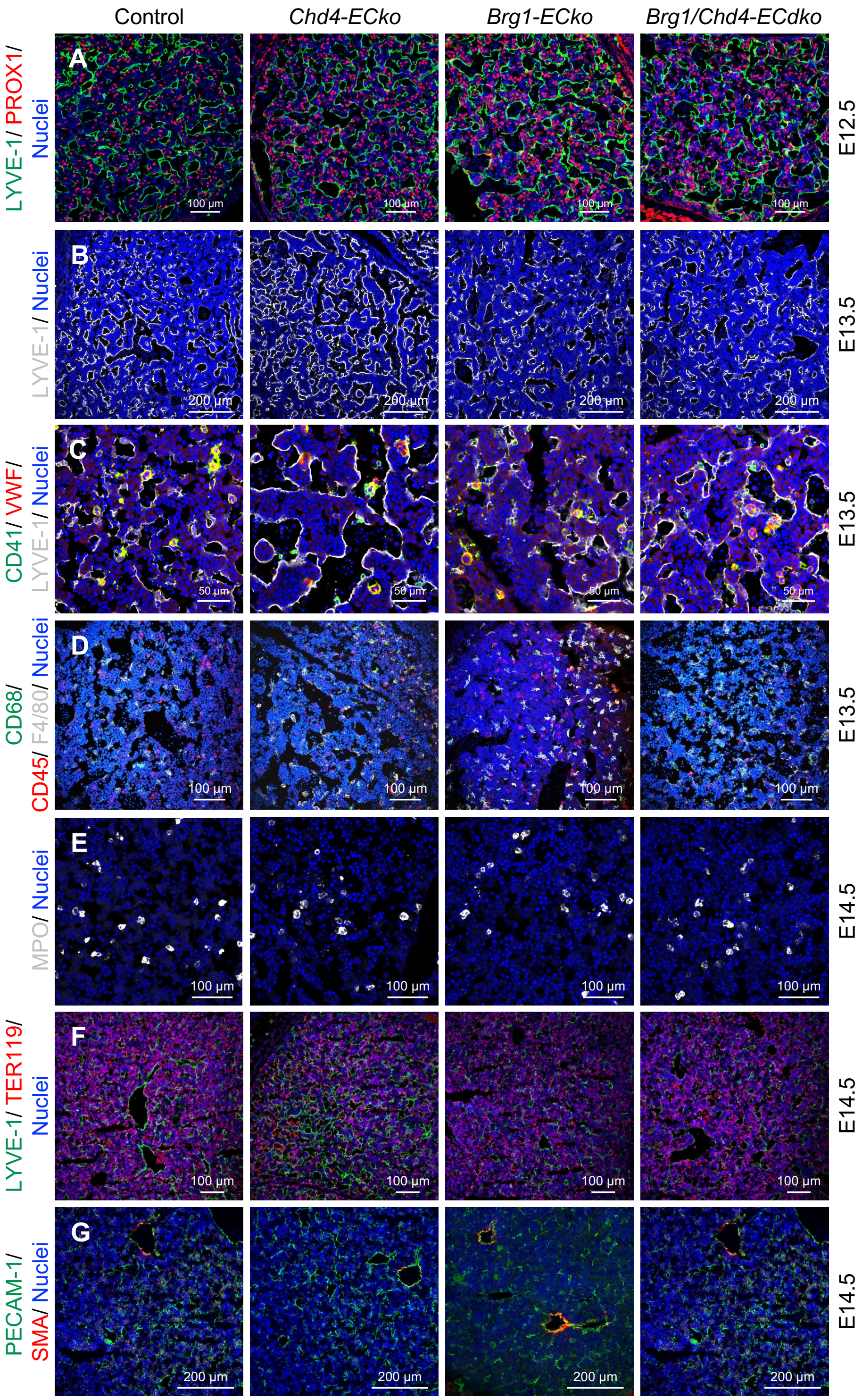

Fig. S6. Immunostaining reveals comparable multicellular environments in control and mutant livers. A. Representative images of immunostaining for LYVE1 (sinusoidal ECs) and PROX1 (hepatoblasts) in control and mutant livers at E12.5. **B.** Representative LYVE1 immunostaining of control and mutant liver sections at E13.5. **C.** Representative immunostaining for CD41 and Von Willebrand factor (VWF) for co-staining of platelets and

Development • Supplementary information

megakaryocytes, and LYVE1 in control and mutant livers at E13.5. Note, VWF also stains some ECs. **D.** Representative immunostaining for CD45 leukocytes, and for CD68 and F4/80 (co-staining for macrophages) in control and mutant livers at E13.5. **E.** Representative immunostaining for myeloperoxidase (MPO) for neutrophils in control and mutant livers at E14.5. **F.** LYVE1 and TER119 (erythrocyte) immunostaining in E14.5 control and mutant livers. **G.** Immunostaining for PECAM1 and for α-smooth muscle actin (SMA) for pericyte and smooth muscle cells in E14.5 control and mutant livers.

Development • Supplementary information

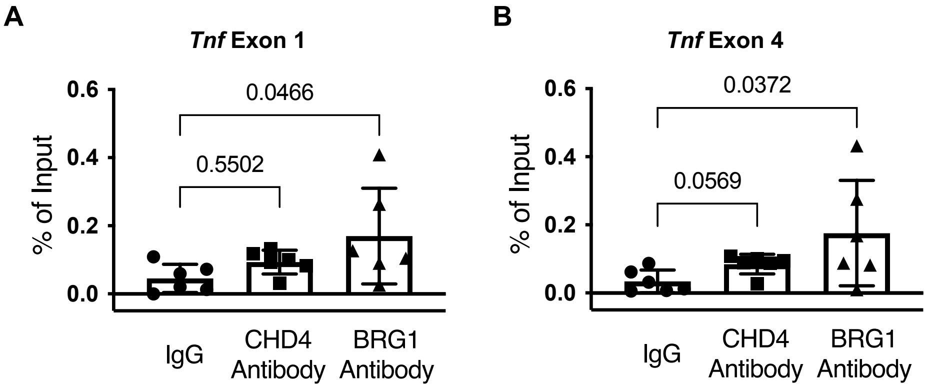

Fig. S7. BRG1 interacts with the *Tnf* gene locus in cultured MS1 cells. ChIP-qPCR was used to determine the enrichment of CHD4 and BRG1 at exon 1 (**A**) and exon 4 (**B**) of the *Tnf* gene locus in MS1 ECs. N=6 independent immunoprecipitations, represented as individual symbols. Data are represented as mean (±s.d.). Ordinary 1-way ANOVA with Dunnett multiple comparisons post hoc tests was used for analysis in A. For B, a Kruskal-Wallis test with Dunn multiple comparisons post hoc test was used due to nonparametric data distribution.

Development • Supplementary information
