# Supplementary material for: Plasmin and sterile inflammation jointly drive fatal embryonic liver degeneration in endothelial chromatin remodeler mutant mice
Source: Development. 2026 May 28;153(16):dev205538. doi: 10.1242/dev.205538 (PMC13286354; doi:10.1242/dev.205538)
Supplement: Supplementary file 1 [file develop-153-205538_review_history.pdf]

Plasmin and sterile inflammation jointly drive fatal embryonic liver degeneration in endothelial chromatin remodeler mutant mice

Meng-Ling Wu, Yelyzaveta Rudenko and Courtney T. Griffin
DOI: 10.1242/dev.205538

Editor: James Wells

Review timeline

Submission to Review Commons:	19 September 2025
Submission to Development:	27 January 2026
Editorial Decision:	24 February 2026
First revision received:	29 April 2026
Accepted:	01 May 2026

Reviewer 1

Evidence, reproducibility and clarity

The manuscript by Wu and Griffin describes a mechanism where CHD4 and BRG1, two chromatin remodelling enzymes, have antagonistic functions to regulate extracellular matrix (ECM) plasmin activity and sterile inflammatory phenotype in the endothelial cells of the developing liver. As a follow up from a previous study, the authors investigate the phenotype of embryonic-lethal endothelial-specific CHD4-knockout, leading to liver phenotype and embryo death, and the rescue of this phenotype when subsequently BRG1 is knocked-out also in the endothelium. First, the authors show that the increase in plasmin activator uPAR (which leads to ECM degradation) in CHD4-KO embryos can be rescued by BRG1-KO, and that both CHD4 and BRG1 interact with the uPAR promoter. However, the authors demonstrate that reducing plasminogen by genetic knockout is unable to rescue the CHD4- KO embryos alone, suggesting an additional mechanism. By RNAseq analysis, the authors identify sterile inflammation as another potential contributor to the lethal phenotype of CHD4- KO embryos through increased expression of ICAM-1 in endothelial cells, also showing binding of both chromatin remodellers to ICAM-1 promoter. Finally, the authors use nonsteroidal anti-inflammatory drug carprofen, alone or in combination with plasminogen genetic knockout, and demonstrate CHD4-KO lethal embryonic phenotype rescue with the combination of plasminogen reduction and inflammation reduction, highlighting the synergistic role of both ECM degradation and sterile inflammation in this genetic KO.

The findings of the manuscript are interesting, experiments well controlled and paper well written. While the work is of potential specialist interest to the field of liver development, there are several issues which authors should address before this paper can be published:

Major issues:

- The authors still see embryonic lethality of some embryos with endothelial BRG1-KO or combined endothelial CHD4/BRG1-KO - could the authors please show or at least comment in the discussion why those animals are dying?
- In the qRT-PCR results Fig.2c, what is each dot?
- In the same figure, I would expect that in CHD4-KO there is no CHD4 transcript, and in BRG1-KO there is no BRG1 transcript, rather than the reduction shown, which seems quite noisy (though significant) - is it this a result of normalisation? Or is indeed only a certain amount of

© 2026. Published by The Company of Biologists under the terms of the Creative Commons Attribution License (https://creativecommons.org/licenses/by/4.0/).

the transcript reduced?
- In the same figure, is the statistical testing performed before or after normalisation? This can introduce errors if done after normalisation.
- In some cases, the authors show immunofluorescence images but do not specify how many biological replicates this represents (e.g. Fig.1d, 4c-d). This should be added.
- I also encourage the authors to present a supplementary figure with at least one other biological replicate shown for imaging data (optional).
- The plasminogen reduction by genetic modulation results in drastic changes to the embryos' appearance - is this a whole embryo KO or endothelial-specific KO? Can authors at least comment on the differences?
- In Fig.2b, do I understand correctly only 1 sample was analysed with different areas plotted on the graph? If so, this experiment should be repeated on another set of embryos to be robust, and data plotted as a mean of each embryo (rather than areas).
- Also in some graphs, authors specify that it was more than n>x embryos, but then – what are the dots on the graph representing? Each embryo? This should be specified (e.g. Fig.2b- c, but please check this in all the figure legends).
- "we found Plaur was the only gene that was induced in CHD4-ECko LSECs at E12.5 (Figure S3D)."
- I am not sure this is correct, as gene Plau is also increased in 2/3 samples?
- I find the title and the running title somewhat misleading and too broad; the authors should specify more detail in the title about the content of the paper - the current statement of the title is somewhat true but shown only for one genetic model and not confirmed for all types of "lethal embryonic liver degeneration".

Minor issues:

- If an animal licence was used, its number should be specified in the ethics or methods section
- In fig.3g it is very hard to see each of the samples, could authors try to improve this graph for clarity using colours-or split Y axis - or both?
- "This indicates that ECs can play a pro-inflammatory role in embryonic livers and highlights the need for tight regulation to ensure normal liver growth." This sentence for me is misleading, EC are producing inflammatory signals only during the CHD4-KO according to the author's data, and authors do not show such data in normal homeostasis condition. Actually, the pro-inflammatory role here seems detrimental, and ECs should not exhibit it for correct development. The authors should rephrase this to be clearer.

Significance

General assessment: The study is well controlled and well written. The findings are interesting. The limitation of the findings is only 1 combination genetic model being studied, and it is unclear if the synergistic effect of sterile inflammation and ECM degradation is broadly applicable to other models, where embryo dies because of liver failure.

Advance: The study makes an incremental advance, following up findings from a previous study. However, it is conceptually interesting.

Audience: The audience for this manuscript would be a liver development specialist. However, broader concepts could also be applicable to liver disease.

Expertise: I research in the field of liver regeneration and disease.

Reviewer 2

Evidence, reproducibility and clarity

In essence, Wu et Al find that Chd4 mutant mice exhibit embryonic liver degeneration due to uPA-mediated plasmin hyperactivity and an ICAM-1-driven hyperinflammation and that additional mutation of BRG1 opposes this liver degeneration, possibly via ICAM-1.

© 2026. Published by The Company of Biologists under the terms of the Creative Commons Attribution License (https://creativecommons.org/licenses/by/4.0/).

Generally, this is an excellent manuscript with a very logical sequence of experiments, although it has shortcomings such as validating their findings in an independent system, ideally human, and further establishing the translational relevance. Establishing translational relevance through mechanistic experiments that identify specific inflammatory tissue pathways, such as by blocking ICAM-1 and TNF-alpha, could also define developmental aberrations as a model for broader (patho)physiology and thereby enhance the impact on the field.

Major

- The embryonic and postnatal survival data of Chd4-ECko and Brg1/Chd4-Ecdko mice should be included in Fig. 1
- What is the impact of Chd4-ECko and Brg1/Chd4-ECdko on the multicellular microenvironment? At a minimum, IF or spatial transcriptomics for hepatocyte and biliary markers, pericytes, and other mesenchymal cells would be recommended. Can there be a distinction made on what type of endothelial cell is affected? (sinusoidal lineage, vs. venous vs. lymphatic)
- The experiments showing how endothelial Chd4 loss leads to a hyperinflammatory endothelial-and potentially hepatoblast-state are important. However, the relevance of immune cell infiltration in the hematopoietic-developing liver remains unclear. Which immune cells are presumably recruited to inflame the microenvironment then? Bone-marrow-derived?

This aspect would benefit from experimental clarification, for example, using migration and/or direct co-culture versus indirect cell co-culture-ideally with or without ICAM-1 blockade-in vitro assays to determine if direct crosstalk with the CD45+ immune cell compartment explains the hyperinflammatory endothelia phenotype.
- Related to the previous comment: Can the authors validate their findings in an independent, ideally human, cell-based system?
- Identifying the specific hematopoietic/immune subset could further increase the paper's impact, as it would more definitively clarify the mechanism in the developing endothelial niche.
- Also, can the authors show experimentally whether, conversely, Chd4 overexpression can limit an endothelial-type of inflammatory liver injury?

Minor

- A separate figure panel for Chd4fl/fl; Vav-Cre+ appears reasonable, instead of being shown as a table.

Significance

Generally, this is an excellent manuscript with a strong developmental biology focus, and its translational relevance is not immediately apparent; however, establishing such a link could significantly increase its impact. For example, the significance of these findings in ischemia-reperfusion injury, SOS/VOD, and sepsis could offer therapeutic avenues to stabilize endothelial function.

The advance is the elegant discovery of a multifactorial endothelial-stabilizing mechanism in development, although its applicability to scenarios beyond developmental mutation remains unknown.

The strengths are the clear and transparent experimental interrogation. Rightfully, the authors acknowledge that there would be a benefit in finalizing inflammatory blockade, genetic or antibody-mediated, to pin down the mechanistic circuit.

The reviewer's expertise is: childhood liver diseases, developmental liver organoid generation, stem cells (iPSCs), cell reprogramming

Reviewer 3

© 2026. Published by The Company of Biologists under the terms of the Creative Commons Attribution License (https://creativecommons.org/licenses/by/4.0/).

Evidence, reproducibility and clarity

Wu et al. report antagonistic roles for chromatin remodelers Chd4 and Brg1, in endothelial cells, during liver development. There is a major flaw in the study which makes it difficult to interpret the conclusions. The genotypes of the mouse models used are flawed. The comparison should be made between two single knockouts (Chd4 single, Brg1 single), double mutants (Chd4/Brg1) and proper controls. For both "single KO", one allele of the other gene is also deleted - Chd4 -Ecko has one allele of Brg1 deleted and vice versa. Also, the proper control should be Chd4 fl/flBrg1fl/fl without the Cre. Since 3 alleles (not just two that belong to the same gene) are deleted in single knockout it is impossible to assign the effect on one gene.

Specific points

1. Fig 2c Plaur transcript - no statistical comparison between 2nd and 4th column, Chd4 Ecko vs double mutant. If there is not statistical difference, does not explain the rescue in double mutants
2. Fig 2e. Comparison should be made between Plg-/- Chd4 fl/fl and Plg-/- Chd4 fl/fl Cre, not other genotypes
3. Fig. 4. How does Chd4 or Brg1 activity in endothelial cells lead to Icam1 activation in epithelial cells?
4. Mice used in Figure 5 are Cdf4 fl/+ and Cdf4 fl/fl, , no Brg1 deletion. The authors improperly compare these to Chd4-Ecko which have one allele of Brg1 deleted. The rescue need s to be done in the same genotype Chd4-Ecko.

Significance

Wu et al. report antagonistic roles for chromatin remodelers Chd4 and Brg1, in endothelial cells, during liver development. There is a major flaw in the study which makes it difficult to interpret the conclusions. Genotypes that were chosen for the study make the data not interpretable

---

Author response to reviewers' comments

1. General Statements [optional]

This section is optional. Insert here any general statements you wish to make about the goal of the study or about the reviews.
This study demonstrates that endothelial CHD4 and BRG1 chromatin remodelers antagonistically regulate transcription of target genes within the plasmin activation and sterile inflammation pathways in the fetal liver. We found that both excessive plasmin activation and inflammation synergistically contribute to liver cell apoptosis and whole liver degeneration in endothelial *Chd4* mutants. This finding is supported by our demonstration that a combination of genetic plasminogen deficiency and pharmacological suppression of inflammation rescues liver phenotypes and lethality in *Chd4*-deficient embryos more effectively than either treatment alone.

These findings provide novel insights into how endothelial transcriptional regulation contributes to hepatic development, linking epigenetic control of extracellular matrix proteolysis and inflammation to liver cell survival. Our work highlights the importance of endothelial cell chromatin remodeling activity in maintaining embryonic liver integrity, which may be relevant to adult liver diseases characterized by excessive plasmin activity and inflammation.

2. Point-by-point description of the revisions

This section is mandatory. Please insert a point-by-point reply describing the revisions that were already carried out and included in the transferred manuscript.

© 2026. Published by The Company of Biologists under the terms of the Creative Commons Attribution License (https://creativecommons.org/licenses/by/4.0/).

Reviewer #1
Evidence, reproducibility and clarity
The manuscript by Wu and Griffin describes a mechanism where CHD4 and BRG1, two chromatin remodelling enzymes, have antagonistic functions to regulate extracellular matrix (ECM) plasmin activity and sterile inflammatory phenotype in the endothelial cells of the developing liver. As a follow up from a previous study, the authors investigate the phenotype of embryonic-lethal endothelial-specific CHD4-knockout, leading to liver phenotype and embryo death, and the rescue of this phenotype when subsequently BRG1 is knocked-out also in the endothelium. First, the authors show that the increase in plasmin activator uPAR (which leads to ECM degradation) in CHD4-KO embryos can be rescued by BRG1-KO, and that both CHD4 and BRG1 interact with the uPAR promoter. However, the authors demonstrate that reducing plasminogen by genetic knockout is unable to rescue the CHD4-KO embryos alone, suggesting an additional mechanism. By RNAseq analysis, the authors identify sterile inflammation as another potential contributor to the lethal phenotype of CHD4-KO embryos through increased expression of ICAM-1 in endothelial cells, also showing binding of both chromatin remodellers to ICAM-1 promoter. Finally, the authors use nonsteroidal anti-inflammatory drug carprofen, alone or in combination with plasminogen genetic knockout, and demonstrate CHD4-KO lethal embryonic phenotype rescue with the combination of plasminogen reduction and inflammation reduction, highlighting the synergistic role of both ECM degradation and sterile inflammation in this genetic KO.

The findings of the manuscript are interesting, experiments well controlled and paper well written. While the work is of potential specialist interest to the field of liver development, there are several issues which authors should address before this paper can be published:

Major issues:

1. The authors still see embryonic lethality of some embryos with endothelial BRG1-KO or combined endothelial CHD4/BRG1-KO - could the authors please show or at least comment in the discussion why those animals are dying?
We observed no dead *Brg1-ECko* or *Brg1/Chd4-ECdko* embryos by E14.5. However, at E17.5, there was an 18.8% lethality rate for *Brg1-ECko* mutants and a 12.5% rate for *Brg1/Chd4- ECdko* mutants (**Fig. 1B**). The reasons behind the incomplete rescue of *Brg1/Chd4-ECdko* embryos and the cause of death in *Brg1-ECko* mutants remain unknown, as we have mentioned in the revised discussion (see lines 311-316).

2. In the qRT-PCR results Fig.2c, what is each dot?
Each dot represents transcripts acquired from a separate embryo. We have modified the figure legend for clarification.

3. In the same figure, I would expect that in CHD4-KO there is no CHD4 transcript, and in BRG1-KO there is no BRG1 transcript, rather than the reduction shown, which seems quite noisy (though significant) - is it this a result of normalisation? Or is indeed only a certain amount of the transcript reduced?
The VE-Cadherin Cre mouse line utilized in this study is reported to have progressive Cre expression and activity from E8.5 to E13.5 and only to reach full penetrance across all vasculature at E14.5[1]. The liver sinusoidal ECs (LSECs) analyzed in **Fig. 2C** were isolated at E12.5, before Cre activity reached its full penetrance. This is likely the primary cause of the variability in gene excision seen in this panel.

4. In the same figure, is the statistical testing performed before or after normalisation? This can introduce errors if done after normalisation.
Normalization was performed before statistical analysis to combine relative transcript counts from embryos harvested in multiple litters. This is now clarified in our methods (see lines 486-489).

5. In some cases, the authors show immunofluorescence images but do not specify how many biological replicates this represents (e.g. Fig.1d, 4c-d). This should be added.
We have updated the legends for **Figs. 1E**, **4C-D**, and **6E-F**, as suggested.

© 2026. Published by The Company of Biologists under the terms of the Creative Commons Attribution License (https://creativecommons.org/licenses/by/4.0/).

6. I also encourage the authors to present a supplementary figure with at least one other biological replicate shown for imaging data (optional).
We appreciated this suggestion but opted not to add additional supplemental figures, which might have been confusing to readers.

7. The plasminogen reduction by genetic modulation results in drastic changes to the embryos' appearance - is this a whole embryo KO or endothelial-specific KO? Can authors at least comment on the differences?
The plasminogen-deficient embryos used in this study were global knockouts; this is now clarified on line 177. The *Chd4-ECko* embryos with varying degrees of plasminogen deficiency that are shown in Fig. 2F were dissected at E17.5, which is ~3 days after the typical time of death for *Chd4-ECko* embryos. This explains why the dead and partially resorbed mutants in **Fig. 2F** look so different from their control ($Plg^{-/-}$) littermate and from the E14.5 *Chd4-ECko* embryos shown in **Fig. 1C**.

8. In Fig.2b, do I understand correctly only 1 sample was analysed with different areas plotted on the graph? If so, this experiment should be repeated on another set of embryos to be robust, and data plotted as a mean of each embryo (rather than areas).
Each dot represents the mean value obtained after quantifying 4 fluorescent areas within a liver section from a single embryo. The N number indicates the number of embryos used from each genotype. We have updated the figure legend accordingly.

9. Also in some graphs, authors specify that it was more than n>x embryos, but then - what are the dots on the graph representing? Each embryo? This should be specified (e.g. Fig.2b-c, but please check this in all the figure legends).
Thank you for this question. We have worked to clarify the legends for all our graphs. Overall, for graphs related to embryos, each dot represents data from a single embryo. Since the sample sizes vary across genotypes, we used the smallest sample size taken from the mutant groups when listing our minimum N.

10. "we found Plaur was the only gene that was induced in CHD4-ECko LSECs at E12.5 (Figure S3D)." - I am not sure this is correct, as gene Plau is also increased in 2/3 samples?
Although *Plau* transcripts were also increased in *Chd4-ECko* LSECs compared to control samples, our statistical analysis showed a p-value of 0.0564, which was deemed non-significant according to our cutoff criteria of $p<0.05$. We have revised our text to emphasize that *Plaur* was the only significantly induced gene in E12.5 LSECs (see line 221).

11. I find the title and the running title somewhat misleading and too broad; the authors should specify more detail in the title about the content of the paper - the current statement of the title is somewhat true but shown only for one genetic model and not confirmed for all types of "lethal embryonic liver degeneration".
We have updated the title to incorporate this suggestion. The revised title is ‘Plasmin activity and sterile inflammation synergize to promote lethal embryonic liver degeneration in endothelial chromatin remodeler mutants.’ The revised running title is ‘Plasmin and inflammation in endothelial mutant livers.’

<u>Minor issues:</u>

12. If an animal licence was used, its number should be specified in the ethics or methods section
We have added this information to the methods (see line 383).

13. In fig.3g it is very hard to see each of the samples, could authors try to improve this graph for clarity using colours-or split Y axis - or both?
We have revised **Fig. 3G** to include a split y-axis, as suggested.

14. "This indicates that ECs can play a pro-inflammatory role in embryonic livers and highlights the need for tight regulation to ensure normal liver growth." This sentence for me is misleading, EC are

© 2026. Published by The Company of Biologists under the terms of the Creative Commons Attribution License (https://creativecommons.org/licenses/by/4.0/).

producing inflammatory signals only during the CHD4-KO according to the author's data, and authors do not show such data in normal homeostasis condition. Actually, the pro- inflammatory role here seems detrimental, and ECs should not exhibit it for correct development. The authors should rephrase this to be clearer.
The detrimental inflammation observed when *Chd4* was deleted in ECs indicates that endothelial CHD4 normally suppresses inflammation during liver development (**Fig. 3F-G**, and **4A-B**). When endothelial CHD4 functions properly, there is no excessive cytokine activation and inflammation. We have modified the sentence to help clarify this information (see lines 295-297).

Significance
General assessment: The study is well controlled and well written. The findings are interesting. The limitation of the findings is only 1 combination genetic model being studied, and it is unclear if the synergistic effect of sterile inflammation and ECM degradation is broadly applicable to other models, where embryo dies because of liver failure.

Advance: The study makes an incremental advance, following up findings from a previous study. However, it is conceptually interesting.

Audience: The audience for this manuscript would be a liver development specialist. However, broader concepts could also be applicable to liver disease.

Expertise: I research in the field of liver regeneration and disease.

Reviewer #2
Evidence, reproducibility and clarity

In essence, Wu et Al find that Chd4 mutant mice exhibit embryonic liver degeneration due to uPA-mediated plasmin hyperactivity and an ICAM-1-driven hyperinflammation and that additional mutation of BRG1 opposes this liver degeneration, possibly via ICAM-1.

Generally, this is an excellent manuscript with a very logical sequence of experiments, although it has shortcomings such as validating their findings in an independent system, ideally human, and further establishing the translational relevance. Establishing translational relevance through mechanistic experiments that identify specific inflammatory tissue pathways, such as by blocking ICAM-1 and TNF-alpha, could also define developmental aberrations as a model for broader (patho)physiology and thereby enhance the impact on the field.

Major

1. The embryonic and postnatal survival data of Chd4-ECko and Brg1/Chd4-Ecdko mice should be included in Fig. 1
We revised Fig. 1 to add representative photos and lethality rates for control and mutant embryos at E17.5 (see new **Fig. 1B**). All *Chd4-ECko* embryos we dissected at E17.5 were dead, which was consistent with our previous report[2]. Although *Brg1/Chd4-ECdko* embryos were largely rescued at E17.5, these mutants still die soon after birth due to lung development issues, as we previously reported[3].

2. What is the impact of Chd4-ECko and Brg1/Chd4-ECdko on the multicellular microenvironment? At a minimum, IF or spatial transcriptomics for hepatocyte and biliary markers, pericytes, and other mesenchymal cells would be recommended. Can there be a distinction made on what type of endothelial cell is affected? (sinusoidal lineage, vs. venous vs. lymphatic)
To assess whether the multicellular microenvironment of *Chd4-ECko* livers was altered, we performed immunostaining for various cellular markers from E12.5 to E14.5. These markers included LYVE-1 for liver sinusoids; PROX1 and E-cadherin (ECAD) for hepatocytes; CD41 for platelets and megakaryocytes; CD45 for leukocytes; CD68 and F4/80 for macrophages; MPO for neutrophils; TER119 for erythroid cells; and a-smooth muscle actin (SMA) for pericytes and smooth muscle cells (see **Fig. 4D** and **Fig. R1**). Across all the images we examined, no obvious cell-type-specific differences were observed between control and mutant livers.

© 2026. Published by The Company of Biologists under the terms of the Creative Commons Attribution License (https://creativecommons.org/licenses/by/4.0/).

Biliary epithelial cells, which begin to differentiate at approximately E15.5[4], were also assessed using cytokeratin 19 (CK19) immunostaining; however, no CK19-positive cells were detected in control livers at E14.5 (see **Fig. R2**). Note that although LYVE-1 is also expressed by lymphatic endothelial cells, lymphatic vessels are not yet established in the liver at E14.5[2]. Therefore, LYVE-1 staining is appropriate for identifying liver sinusoidal ECs at this stage of development. Our data indicate that the affected vasculature in *Chd4-ECko* livers is predominantly localized to the liver periphery (see Fig. 1D), which LYVE-1 staining shows to be mostly populated by sinusoidal vessels (**Fig. R1B and R1F**).

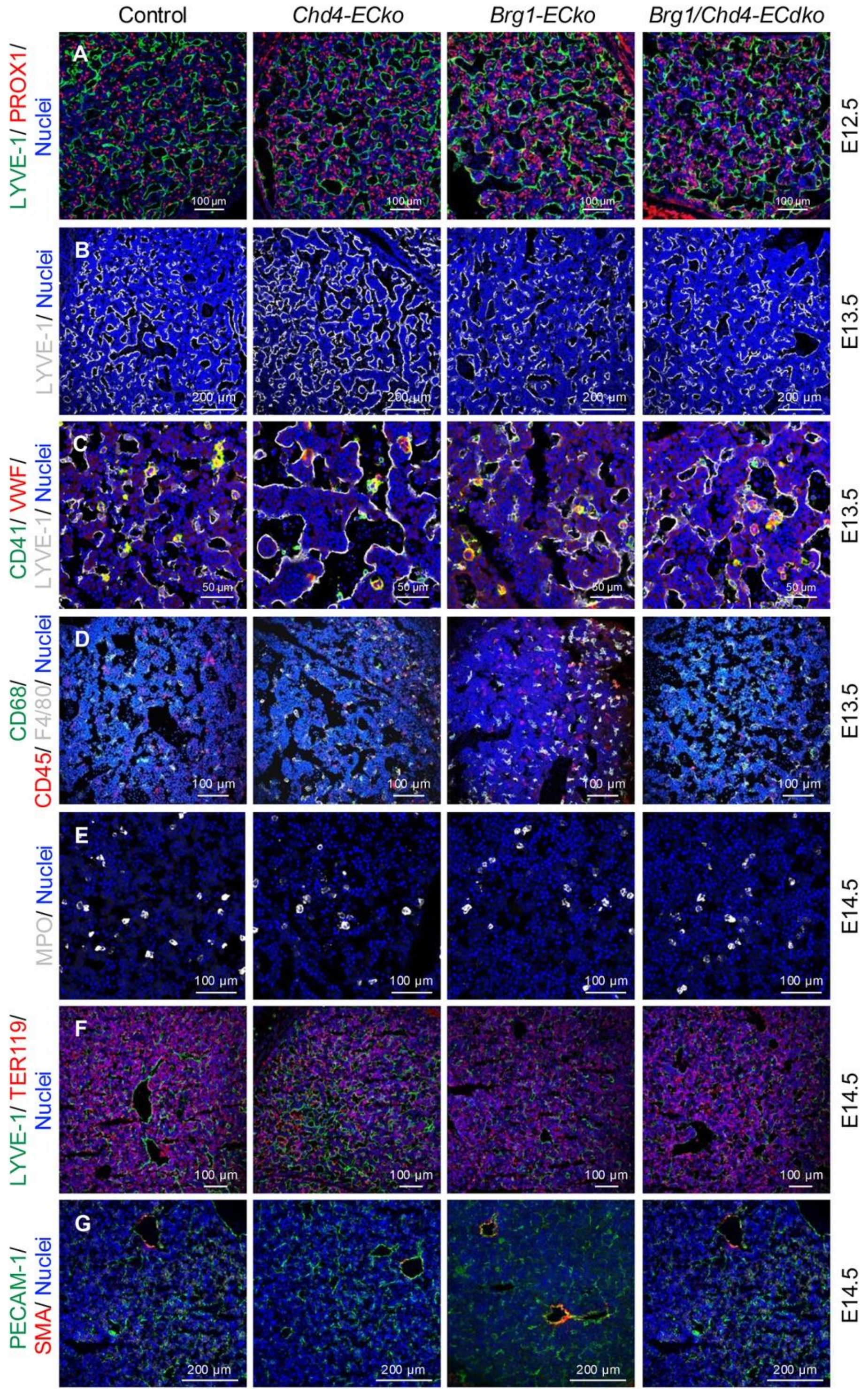

Fig. R1: Immunostaining reveals a similar multicellular environment in control and mutant

© 2026. Published by The Company of Biologists under the terms of the Creative Commons Attribution License (https://creativecommons.org/licenses/by/4.0/).

livers. Liver sections from E12.5 to E14.5 control and mutant embryos were assessed by staining for multiple cell markers: LYVE-1 for liver sinusoids; PROX1 and E-cadherin (ECAD) for hepatocytes; CD41 for platelets and megakaryocytes; CD45 for leukocytes; CD68 and F4/80 for macrophages; MPO for neutrophils; TER119 for erythroid cells; and a-smooth muscle actin (SMA) for pericytes and smooth muscle cells.

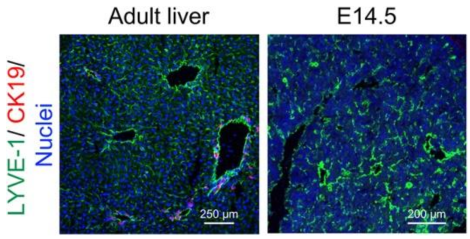

Fig. R2: Immunostaining of CK19, a biliary epithelial cell marker, in adult and E14.5 livers. Liver sections were stained for CK19, which marks biliary epithelial cells, and LYVE-1, which labels liver sinusoids. No biliary epithelial cells were visible in the E14.5 liver sections we analyzed.

3. The experiments showing how endothelial Chd4 loss leads to a hyperinflammatory endothelial- and potentially hepatoblast-state are important. However, the relevance of immune cell infiltration in the hematopoietic-developing liver remains unclear. Which immune cells are presumably recruited to inflame the microenvironment then? Bone-marrow-derived? This aspect would benefit from experimental clarification, for example, using migration and/or direct co-culture versus indirect cell co-culture-ideally with or without ICAM-1 blockade-in vitro assays to determine if direct crosstalk with the CD45+ immune cell compartment explains the hyperinflammatory endothelia phenotype.

In mice, the first hematopoietic cells emerge in the yolk sac at E7.5[5]. Subsequently, embryonic hematopoiesis takes place in the aorta-gonad-mesonephros (AGM) region and the placenta, before immature hematopoietic cells migrate to the fetal liver. After E11.0, the fetal liver becomes the main hematopoietic organ, supporting the expansion and differentiation of hematopoietic stem and progenitor cells into all mature blood cell lineages[5-8]. Around E16.5, hematopoietic cells migrate to the bone marrow[9], so the bone marrow is not a relevant source of infiltrating immune cells in our E12.5-14.5 *Chd4-ECko* mutants. We therefore examined immune cell populations, including leukocytes, macrophages, and neutrophils, in *Chd4-ECko* livers. No enrichment of specific immune cell types was observed in *Chd4-ECko* livers compared with controls at E13.5-14.5 (**Fig. R1**). Since immune cells develop within fetal livers at this stage, these findings suggest that they are locally activated rather than recruited to *Chd4-ECko* livers. Moreover, because fetal livers contain a heterogeneous mixture of immature and mature hematopoietic and immune cells, appropriate in vitro cell models to assess immune cell activation in this context are currently lacking. We have added comments to the introduction to address some of these points (see lines 66-68).

4. Related to the previous comment: Can the authors validate their findings in an independent, ideally human, cell-based system?
To explore this, we analyzed *PLAUR* and *ICAM1* transcripts following CHD4 and/or BRG1 knockdown in primary human umbilical vein endothelial cells (HUVECs) for 48 hours. No antagonistic regulation of either gene was detected in HUVECs (**Fig. R3**). Moreover, while *Icam1* transcription was antagonistically regulated by CHD4 and BRG1 in the mouse MS1 EC line (see **Fig. 5A**), transcriptional regulation of *Plaur* by these remodelers was observed only in isolated LSECs and not in cultured MS1 cells. Together, these findings demonstrate that BRG1 and CHD4 play context-specific roles when regulating *Icam1* and *Plaur* transcription in different EC types. Furthermore, in vitro versus in vivo EC environments may additionally influence BRG1 and CHD4 activity.

© 2026. Published by The Company of Biologists under the terms of the Creative Commons Attribution License (https://creativecommons.org/licenses/by/4.0/).

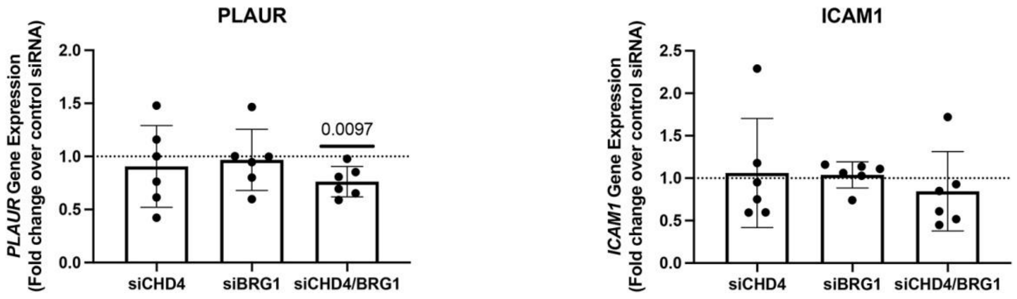

Fig. R3: Transcripts of *PLAUR* and *ICAM1* following knockdown of CHD4 and/or BRG1 in HUVECs. qRT-PCR analysis showing *PLAUR* and *ICAM1* gene transcripts in HUVECs at 48 hours after knockdown. The dotted line represents the relative expression of transcripts after treatment with a control siRNA.

5. Identifying the specific hematopoietic/immune subset could further increase the paper's impact, as it would more definitively clarify the mechanism in the developing endothelial niche.
Please see our response to question # 3.

6. Also, can the authors show experimentally whether, conversely, Chd4 overexpression can limit an endothelial-type of inflammatory liver injury?
We agree that exploring this suggestion would provide useful insights. However, we currently lack a genetic or inducible endothelial-specific *Chd4* overexpression model, which makes it challenging to link our embryonic findings to the context of adult liver injury. For now, our study demonstrates that hepatic ECs regulate sterile inflammation to support embryonic liver development. Future development of appropriate genetic tools will allow us to determine if the role of endothelial CHD4 that is demonstrated in the current study is recapitulated in adult inflammatory liver injury models.

<u>Minor</u>

7. A separate figure panel for Chd4fl/fl; Vav-Cre+ appears reasonable, instead of being shown as a table.
Thank you. Please see our new **Fig. S1**, which includes representative images (and lethality rates) of control and *Chd4*$^{fl/fl}$;*Vav-Cre*$^{+}$ embryos at E18.5.

Significance:

Generally, this is an excellent manuscript with a strong developmental biology focus, and its translational relevance is not immediately apparent; however, establishing such a link could significantly increase its impact. For example, the significance of these findings in ischemia-reperfusion injury, SOS/VOD, and sepsis could offer therapeutic avenues to stabilize endothelial function.

The advance is the elegant discovery of a multifactorial endothelial-stabilizing mechanism in development, although its applicability to scenarios beyond developmental mutation remains unknown.

The strengths are the clear and transparent experimental interrogation. Rightfully, the authors acknowledge that there would be a benefit in finalizing inflammatory blockade, genetic or antibody-mediated, to pin down the mechanistic circuit.

The reviewer's expertise is: childhood liver diseases, developmental liver organoid generation, stem cells (iPSCs), cell reprogramming

Reviewer #3
Evidence, reproducibility and clarity:

1. Wu et al. report antagonistic roles for chromatin remodelers Chd4 and Brg1, in endothelial

© 2026. Published by The Company of Biologists under the terms of the Creative Commons Attribution License (https://creativecommons.org/licenses/by/4.0/).

cells, during liver development. There is a major flaw in the study which makes it difficult to interpret the conclusions. The genotypes of the mouse models used are flawed. The comparison should be made between two single knockouts (Chd4 single, Brg1 single), double mutants (Chd4/Brg1) and proper controls. For both "single KO", one allele of the other gene is also deleted - Chd4 -Ecko has one allele of Brg1 deleted and vice versa. Also, the proper control should be Chd4 fl/flBrg1fl/fl without the Cre. Since 3 alleles (not just two that belong to the same gene) are deleted in a single knockout, it is impossible to assign the effect to one gene.
We acknowledge the fact that the single *Brg1* and *Chd4* EC knockouts in this study each carry a heterozygous deletion allele for the other remodeler (exact genotypes are shown in Fig. 1A). The mating strategy that yielded these mutants was chosen for three reasons. *First*, we have found that genetic background influences the embryonic phenotypes of these chromatin remodeler mutants[3]. Moreover, embryonic development at the stages analyzed in this study occurs quickly and requires precise timing for comparative analysis between genotypes. Therefore, it is most rigorous to study littermates when comparing single- and double-mutant embryos for BRG1 and CHD4. To achieve this, we used $Brg1^{fl/fl}$;$Chd4^{fl/fl}$ females rather than $Brg1^{fl/+}$;$Chd4^{fl/+}$ females for timed matings. Although the former females cannot produce single knockout embryos without a compound heterozygous allele of the other remodeler, these females allowed us to generate single- and double-knockouts at a rate of 1/8 embryos. If we had used $Brg1^{fl/+}$;$Chd4^{fl/+}$ females for timed matings, we would have been able to generate “clean” single mutants with wildtype alleles of the other remodeler, but the single- and double- knockout generation rate would have been 1/32 embryos. This would have been an impractical mutant generation rate for this study. *Second*, our prior research demonstrates that heterozygous deletion of *Chd4* or *Brg1* does not produce the liver phenotypes seen with the respective homozygous deletions[2,3]. *Third*, the complete lethality of *Chd4-ECko* ($Brg1^{fl/+}$;$Chd4^{fl/fl}$;*VE-cadherin-Cre*$^{+}$) mutants in this study demonstrates that deleting one allele of *Brg1* cannot rescue *Chd4*-related lethality.

As for controls in this study, we saw no evidence of phenotypes or of any gene deletion in our *Cre*$^{-}$ embryos (either in this study or in previous ones analyzing similar phenotypes[2,3]). Therefore, we used *Cre*$^{-}$ embryos for controls because they were generated at a 1/2 rate by our timed matings, which boosted our output for analyses.

Specific points
2. Fig 2c Plaur transcript - no statistical comparison between 2nd and 4th column, Chd4 Ecko vs double mutant. If there is not statistical difference, does not explain the rescue in double mutants
Thank you for the suggestion. We have included a comparison between *Chd4-ECko* and *Brg1/Chd4-ECdko* in our revised **Fig 2C**. The Kruskal-Wallis test showed a significant difference between the *Chd4-Ecko* and *Brg1/Chd4-ECdko* groups (p=0.016). This indicates that *Plaur* induction in *Chd4-Ecko* LSECs is rescued in *Brg1/Chd4-ECdko* LSECs.

3. Fig 2e. Comparison should be made between Plg-/- Chd4 fl/fl and Plg-/- Chd4 fl/fl Cre, not other genotypes
This experiment aims to determine whether different levels of plasminogen (*Plg*) reduction can rescue the lethality caused by *Chd4* deletion. To do this, we set up the mating strategy shown in **Fig. 2E** to produce appropriate littermate controls and to compare lethality among $Plg^{+/+}$;*Chd4-ECko*, $Plg^{+/-}$;*Chd4-ECko*, and $Plg^{-/-}$;*Chd4-ECko* embryos. This comparison would not have been possible with embryos generated only from mice on a $Plg^{-/-}$ background.

4. Fig. 4. How does Chd4 or Brg1 activity in endothelial cells lead to Icam1 activation in epithelial cells?
Since cytokines like IFNg, TNFa, and IL1b can induce ICAM-1 expression in hepatocytes[10], we speculate that ICAM-1 expression in hepatoblasts (ECAD^{+} cells in **Fig. 4D**) was induced by the elevated TNFa and IL1b produced in *Chd4-ECko* livers (**Fig. 3G**).

© 2026. Published by The Company of Biologists under the terms of the Creative Commons Attribution License (https://creativecommons.org/licenses/by/4.0/).

5. Mice used in Figure 5 are Cdf4 fl/+ and Cdf4 fl/fl, no Brg1 deletion. The authors improperly compare these to Chd4-Ecko which have one allele of Brg1 deleted. The rescue needs to be done in the same genotype Chd4-Ecko.
Please note that data from **Fig. 5** were generated from cultured ECs (MS1 cells).

Significance

Wu et al. report antagonistic roles for chromatin remodelers Chd4 and Brg1, in endothelial cells, during liver development. There is a major flaw in the study which makes it difficult to interpret the conclusions. Genotypes that were chosen for the study make the data not interpretable

---

In summary, we have included the following changes to this revised manuscript:

- **New Figure 1B:** Representative images and lethality rates for control, *Chd4-ECko*, *Brg1-ECko*, and *Brg1/Chd4-ECdko* embryos at E17.5.
- **New Figure 2C:** qRT-PCR analysis of *Chd4*, *Brg1*, and *Plaur* gene transcripts in E12.5 control and mutant LSECs.
- **Regraphing of Figure 3G:** qRT-PCR analysis of *Tnf*, *Il6*, and *Il1b* gene transcripts in E14.5 control and mutant livers.
- **New Figure S1:** Representative images and lethality rates for control, $Chd4^{fl/+}$;*Vav-Cre*$^{+}$, and $Chd4^{fl/fl}$;*Vav-Cre*$^{+}$ embryos at E18.5.

References for this revision:

1. Alva JA, Zovein AC, Monvoisin A, Murphy T, Salazar A, Harvey NL, Carmeliet P, Iruela-Arispe ML. VE-Cadherin-Cre-recombinase Transgenic Mouse: A Tool for Lineage Analysis and Gene Deletion in Endothelial Cells. *Dev Dyn*. 2006;235:759-767. doi: 10.1002/dvdy.20643
2. Crosswhite PL, Podsiadlowska JJ, Curtis CD, Gao S, Xia L, Srinivasan RS, Griffin CT. CHD4-regulated plasmin activation impacts lymphovenous hemostasis and hepatic vascular integrity. *J Clin Invest*. 2016;126:2254-2266. doi: 10.1172/JCI84652
3. Wu ML, Wheeler K, Silasi R, Lupu F, Griffin CT. Endothelial Chromatin-Remodeling Enzymes Regulate the Production of Critical ECM Components During Murine Lung Development. *Arterioscler Thromb Vasc Biol*. 2024;44:1784-1798. doi: 10.1161/ATVBAHA.124.320881
4. Shiojiri N, Inujima S, Ishikawa K, Terada K, Mori M. Cell lineage analysis during liver development using the spf^{ash}-heterozygous mouse. *Lab Invest*. 2001;81:17-25. doi: 10.1038/labinvest.3780208
5. Soares-da-Silva F, Peixoto M, Cumano A, Pinto-do OP. Crosstalk Between the Hepatic and Hematopoietic Systems During Embryonic Development. *Front Cell Dev Biol*. 2020;8:612. doi: 10.3389/fcell.2020.00612
6. Ema H, Nakauchi H. Expansion of hematopoietic stem cells in the developing liver of a mouse embryo. *Blood*. 2000;95:2284-2288.
7. Kieusseian A, Brunet de la Grange P, Burlen-Defranoux O, Godin I, Cumano A. Immature hematopoietic stem cells undergo maturation in the fetal liver. *Development*. 2012;139:3521-3530. doi: 10.1242/dev.079210
8. Freitas-Lopes MA, Mafra K, David BA, Carvalho-Gontijo R, Menezes GB. Differential Location and Distribution of Hepatic Immune Cells. *Cells*. 2017;6. doi: 10.3390/cells6040048
9. Christensen JL, Wright DE, Wagers AJ, Weissman IL. Circulation and chemotaxis of fetal hematopoietic stem cells. *PLoS Biol*. 2004;2:E75. doi: 10.1371/journal.pbio.0020075
10. Satoh S, Nussler AK, Liu ZZ, Thomson AW. Proinflammatory cytokines and endotoxin stimulate ICAM-1 gene expression and secretion by normal human hepatocytes. *Immunology*. 1994;82:571-576.

© 2026. Published by The Company of Biologists under the terms of the Creative Commons Attribution License (https://creativecommons.org/licenses/by/4.0/).

Original submission

First decision letter

MS ID#: dev.205538

MS Title: Plasmin activity and sterile inflammation synergize to promote lethal embryonic liver degeneration

Authors: Meng-Ling Wu; Yelyzaveta Rudenko; Courtney Griffin

Dear Dr Griffin,

Thank you for sending your manuscript to Development through Review Commons.

I have now received all the referees reports on the above manuscript, and have reached a decision. The referees' comments are appended below.

The overall evaluation is positive and we would like to publish a revised manuscript in Development, provided that the referees' comments can be satisfactorily addressed. In particular, reviewer 3 has some suggestion for revising your manuscript, one relating to comparison with human liver, or being more clear that this is a mouse-specific. Reviewer 1 remains concerned about the genotype of the control and experimental animals. Your response seems to address this, so I suggest including this potential concern in the discussion. Please include with your revised manuscript point-by-point response. If you do not agree with any of their criticisms or suggestions explain clearly why this is so. If it would be helpful, you are welcome to contact us to discuss your revision in greater detail. Please send us a point-by-point response indicating your plans for addressing the referees' comments, and we will look over this and provide further guidance.

Reviewer 1

SUMMARY OF THE ADVANCE MADE IN THIS PAPER AND ITS POTENTIAL SIGNIFICANCE TO THE FIELD

As started in the first review, use of genetic models where full deletion of one gene and half of the other are called single KO is not proper genetic analysis and makes it difficult to interpret the results. In addition, if genetic background influences the phenotype, how robust is the phenotype? ? If the phenotype is not robust and is only present in one strain of mice, instead of diverse outbred population, the results are not as relevant.

SUGGESTIONS TO AUTHORS

Use proper genetic models and accurate statistical analysis.
Please see comments in the attached document

Major Point

Unfortunately, the reviewers chose not to address my concerns about genetic models used. This is a major flaw in the study still makes it difficult to interpret the conclusions. In responses, authors cite ease of experiments and analyses as the reason for matings and genotypes chosen. That is not a valid alternative to use of proper genetic models to test their hypothesis. Also, statistical analysis is not done correctly in several instances in the manuscript.

“We acknowledge the fact that the single Brg1 and Chd4 EC knockouts in this study each carry a heterozygous deletion allele for the other remodeler (exact genotypes are shown in Fig. 1A). The mating strategy that yielded these mutants was chosen for three reasons. First, we have found that genetic background influences the embryonic phenotypes of these chromatin remodeler mutants”

© 2026. Published by The Company of Biologists under the terms of the Creative Commons Attribution License (https://creativecommons.org/licenses/by/4.0/).

First, my concern about lack of real single mutants stands is not addressed. Also, if genetic background influences the phenotype, how robust is the phenotype? The paper they cited was embryonic phenotype in the lung. Does it work the same way in the liber? If the phenotype is not robust and is only present in one strain of mice, instead of diverse outbred population, the results are not as relevant. Also, reference to their mating scheme is not a valid argument. You do not have to generate all the embryos from the same mating. Single mutants should be generated independently.

"Our prior research demonstrates that heterozygous deletion of Chd4 or Brg1 does not produce the liver phenotypes seen with the respective homozygous deletions"

References cited do not prove this point. The first one is a deletion in the lung. The second reports that single Chd4 KO (cadherin Cre) are embryonic lethal

"Therefore, we used Cre- embryos for controls because they were generated at a 1/2 rate by our timed matings, which boosted our output for analyses"

Ease of experiment or analysis is not a valid argument. Accepted genetic controls are littermates, with the same floxed alleles . Those without Cre are called WT, those with Cre are mutants. While insertion of LoxP cites is not expected to have an effect, if it does, a proper genetic comparison eliminates this confound variable.

<u>Specific points</u>

2. Fig 2c Plaur transcript - no statistical comparison between 2nd and 4th column, Chd4 Ecko vs double mutant. If there is not statistical difference, does not explain the rescue in double mutants

"Thank you for the suggestion. We have included a comparison between Chd4-ECko and Brg1/Chd4-ECdko in our revised Fig 2C. The Kruskal-Wallis test showed a significant difference"

T-test is usually used to analyze changes in gene expression. Not clear why non-parametric Kruskal-Wallis test was chosen here.

3. Fig 2e. Comparison should be made between Plg-/- Chd4 fl/fl and Plg-/- Chd4 fl/fl Cre, not other genotypes

"This experiment aims to determine whether different levels of plasminogen (Plg) reduction can rescue the lethality caused by Chd4 deletion. To do this, we set up the mating strategy shown inFig. 2E to produce appropriate littermate controls and to compare lethality among Plg+/+;Chd4-ECko, Plg+/-;Chd4-ECko, and Plg-/-;Chd4-ECko embryos. This comparison would not have been possible with embryos generated only from mice on a Plg-/- background."

The authors want to determine " whether different levels of plasminogen (Plg) reduction can rescue the lethality caused by Chd4 deletion." Again, Chd4 deletion is compounded by loss of one allele of Brg1 in their model

4. Fig. 4. How does Chd4 or Brg1 activity in endothelial cells lead to Icam1 activation in epithelial cells?

Since cytokines like IFNg, TNFa, and IL1b can induce ICAM-1 expression in hepatocytes10, we speculate that ICAM-1 expression in hepatoblasts (ECAD+ cells in Fig. 4D) was induced by the elevated TNFa and IL1b produced in Chd4-ECko livers (Fig. 3G).

No mechanism tested, just speculation

5. Mice used in Figure 5 are Cdf4 fl/+ and Cdf4 fl/fl, no Brg1 deletion. The authors improperly compare these to Chd4-Ecko which have one allele of Brg1 deleted. The rescue needs to be done in the same genotype Chd4-Ecko.

© 2026. Published by The Company of Biologists under the terms of the Creative Commons Attribution License (https://creativecommons.org/licenses/by/4.0/).

“Please note that data from Fig. 5 were generated from cultured ECs (MS1 cells).”

Why does siBRG1 affect expression of Chd4 in f5A? Also, if Chd4 and Brg1 work in different direction, why is Icam1 expression elevated in double mutants as compared to siControl. Again, statistics are not done properly, no comparison between column 1 (siControl) and column 4 (siBRG1/Chr4)

<u>Reviewer 2</u>

My comments were sufficiently addressed, and I have no further comments.

<u>Reviewer 3</u>

The authors have addressed many points, but some clarification is necessary.

1. Considering the IF images presented in R1 (which should be incorporated into the manuscript rather than solely as a rebuttal figure), could the developing sinusoids perhaps be marginally wider in the Chd4-KO condition? This consideration might be significant as it could influence hemodynamics within the rapidly expanding liver. A comparison with experimental measurements appears relevant to me, along with a discussion of its potential relevance to the peripheral bleeding phenotype. Does it promote blood leakage, or suggest impaired endothelial barrier stability and/or extracellular matrix/capsule integrity?
2. Since no antagonistic effect was observed in HUVECs, external datasets of human LSEC-like cells derived from iPSCs, alone or in organoids, could be assessed and for Chd4 expression in human settings, e.g. https://www.nature.com/articles/s41598-024-64195-1, or https://www.sciencedirect.com/science/article/pii/S2589555924000247 . At any rate, a revised title should reflect that the paper's observation relates to mice.
3. Minor: the discussion should be updated to note that models of the fetal liver, including a heterogeneous mixture of immune cells, albeit in their first iterations, do exist, https://pubmed.ncbi.nlm.nih.gov/41418844/
4. Minor: A discussion should be provided regarding whether subsets of macrophages may be involved in the rescue phenotype of the double-KO-mice. This is relevant because the staining in R1 does not permit elucidation as to whether specific repair or anti-inflammatory subsets of yolk sac-derived macrophages are involved in mitigating the repair.

First revision

<u>Author response to reviewers' comments</u>

Reviewer 1:

<u>Major Point</u>
1. Unfortunately, the reviewers chose not to address my concerns about genetic models used. This is a major flaw in the study still makes it difficult to interpret the conclusions. In responses, authors cite ease of experiments and analyses as the reason for matings and genotypes chosen. That is not a valid alternative to use of proper genetic models to test their hypothesis. Also, statistical analysis is not done correctly in several instances in the manuscript. [*from the Reviewer’s online summary:* As started in the first review, use of genetic models where full deletion of one gene and half of the other are called single KO is not proper genetic analysis and makes it difficult to interpret the results. In addition, if genetic background influences the phenotype, how robust is the phenotype? ? If the phenotype is not robust and is only present in one strain of mice, instead of diverse outbred population, the results are not as relevant.]

“We acknowledge the fact that the single Brg1 and Chd4 EC knockouts in this study each carry a heterozygous deletion allele for the other remodeler (exact genotypes are shown in Fig. 1A).

© 2026. Published by The Company of Biologists under the terms of the Creative Commons Attribution License (https://creativecommons.org/licenses/by/4.0/).

The mating strategy that yielded these mutants was chosen for three reasons. First, we have found that genetic background influences the embryonic phenotypes of these chromatin remodeler mutants”

First, my concern about lack of real single mutants stands is not addressed. Also, if genetic background influences the phenotype, how robust is the phenotype? The paper they cited was embryonic phenotype in the lung. Does it work the same way in the liber? If the phenotype is not robust and is only present in one strain of mice, instead of diverse outbred population, the results are not as relevant. Also, reference to their mating scheme is not a valid argument. You do not have to generate all the embryos from the same mating. Single mutants should be generated independently.

“Our prior research demonstrates that heterozygous deletion of Chd4 or Brg1 does not produce the liver phenotypes seen with the respective homozygous deletions”

References cited do not prove this point. The first one is a lung deletion. The second reports that single Chd4 KO (cadherin Cre) are embryonic lethal.

We acknowledge the Reviewer’s continued concerns about the genetic mutants and controls generated for this study. While we still believe that our genetic approach is sound, for the reasons detailed in our previous rebuttal letter, we have now added a “Study Limitations” section to the end of the “Discussion” to address these concerns with transparency (see lines 416-421). We have also highlighted this point in the “Results” (see lines 135-137).

In terms of the specific concern about the *Chd4* mutant liver phenotype being sensitive to background and therefore not robust, we want to clarify that the *Chd4* mutant liver phenotype was first observed on mixed background strains[1], where it resulted in 100% lethality by E16.5. In the current study, which was conducted 10 years later, we observed similar timing of lethality on a mixed background (100% lethality by E17.5), and this timing was maintained on an outbred CD1 background. However, when we crossed the *Chd4* mutant line to an inbred C57Bl/6J background, some mutant embryos survived longer than E17.5 (we did not do a thorough analysis of whether and how long they lived after birth). Therefore, while the *Chd4* mutant liver phenotype is variable on an inbred C57Bl/6J background, other mixed and outbred background show a consisting timing and penetrance of the liver phenotype.

Finally, the two references we provided in our previous rebuttal (PMC4887170 and PMC11624602[1,2]) were both intended to demonstrate that *Chd4*$^{fl/+}$*;VE-Cadherin-Cre*$^{+}$ embryos do not exhibit liver phenotypes or embryonic lethality. Furthermore, *Brg1*$^{fl/fl}$*;VE-Cadherin-Cre*$^{+}$ embryos survive past birth without obvious vascular phenotypes (see also PMC4579958[3]).

“Therefore, we used Cre- embryos for controls because they were generated at a 1/2 rate by our timed matings, which boosted our output for analyses”

Ease of experiment or analysis is not a valid argument. Accepted genetic controls are littermates, with the same floxed alleles . Those without Cre are called WT, those with Cre are mutants. While insertion of LoxP cites is not expected to have an effect, if it does, a proper genetic comparison eliminates this confound variable.

When available, we selected *Brg1*$^{fl/fl}$*;Chd4*$^{fl/fl}$ embryos (from the same litter as mutants) as controls. In litters that did not contain these controls, we used *Brg1*$^{fl/+}$*;Chd4*$^{fl/fl}$ embryos, *Brg1*$^{fl/fl}$*;Chd4*$^{fl/+}$ embryos, or *Brg1*$^{fl/+}$*;Chd4*$^{fl/+}$ embryos as controls. All these control genotypes possess at least one floxed allele for both *Brg1* and *Chd4*. This point is highlighted in our “Limitations section” (see lines 421-424).

1. Fig 2c Plaur transcript - no statistical comparison between 2nd and 4th column, Chd4 Ecko vs double mutant. If there is not statistical difference, does not explain the rescue in double mutants

“Thank you for the suggestion. We have included a comparison between Chd4-ECko and Brg1/Chd4-ECdko in our revised Fig 2C. The Kruskal-Wallis test showed a significant difference”

T-test is usually used to analyze changes in gene expression. Not clear why non-parametric Kruskal-Wallis test was chosen here.

Since we performed multiple comparisons among these groups (Control vs. *Chd4-ECko* comparison, Control vs. *Brg1-ECko* comparison, and *Chd4-ECko* vs. *Brg1/Chd4-ECko* comparison), we opted to perform a one-way ANOVA rather than a t-test for statistical analysis. The one-way ANOVA reduces the chance of false positives from multiple comparisons. The non-parametric Kruskal-Wallis test combined with Dunn’s was selected as a post hoc test because the data failed the normality test (D’Agostino-Pearson omnibus).

© 2026. Published by The Company of Biologists under the terms of the Creative Commons Attribution License (https://creativecommons.org/licenses/by/4.0/).

2. Fig 2e. Comparison should be made between Plg-/- Chd4 fl/fl and Plg-/- Chd4 fl/fl Cre, not other genotypes

"This experiment aims to determine whether different levels of plasminogen (Plg) reduction can rescue the lethality caused by Chd4 deletion. To do this, we set up the mating strategy shown in Fig. 2E to produce appropriate littermate controls and to compare lethality among Plg+/+;Chd4- ECko, Plg+/-;Chd4-ECko, and Plg-/-;Chd4-ECko embryos. This comparison would not have been possible with embryos generated only from mice on a Plg-/- background."

The authors want to determine "whether different levels of plasminogen (Plg) reduction can rescue the lethality caused by Chd4 deletion." Again, Chd4 deletion is compounded by loss of one allele of Brg1 in their model

This experiment attempted to compare the effects of different levels of *Plg* on *Chd4* mutant lethality. We set up matings between *Plg*$^{+/-}$;*Chd4*$^{fl/+}$;*VE-cadherin-Cre*$^{+}$ male mice and *Plg*$^{+/-}$;*Chd4*$^{fl/fl}$ female mice to generate the desired embryos. Just to clarify, *Chd4* mutants did *not* carry one floxed allele of *Brg1* in this experiment. To reduce confusion for readers, we have now discarded all the shorthand names used in the text related to these *Plg* deficiency experiments (see pgs. 9-14) and in **Figs. 2F**, **6B-F**, and **S3A**.

3. Fig. 4. How does Chd4 or Brg1 activity in endothelial cells lead to Icam1 activation in epithelial cells?

"Since cytokines like IFNg, TNFa, and IL1b can induce ICAM-1 expression in hepatocytes10, we speculate that ICAM-1 expression in hepatoblasts (ECAD+ cells in Fig. 4D) was induced by the elevated TNFa and IL1b produced in Chd4-ECko livers (Fig. 3G)."

No mechanism tested, just speculation

Our hypothesis that TNF-α and IL-1β generated from *Chd4* mutant endothelial cells drive the upregulation of ICAM-1 in hepatoblasts is based on reports that these cytokines can induce ICAM-1 expression in cultured hepatocytes[4,5]. To test this hypothesis in vivo, we would need to cross floxed alleles of *Tnf* and *Il1b* onto our *Chd4-ECko* mutant line and determine if ICAM-1 was no longer upregulated on hepatoblasts as it is in Fig. 4D. We currently lack the mouse models to perform this experiment, and it would take well over a year to acquire these models and conduct the necessary breeding to address this single question.

4. Mice used in Figure 5 are Cdf4 fl/+ and Cdf4 fl/fl, no Brg1 deletion. The authors improperly compare these to Chd4-Ecko which have one allele of Brg1 deleted. The rescue needs to be done in the same genotype Chd4-Ecko.

"Please note that data from Fig. 5 were generated from cultured ECs (MS1 cells)."

Why does siBRG1 affect expression of Chd4 in f5A? Also, if Chd4 and Brg1 work in different direction, why is Icam1 expression elevated in double mutants as compared to siControl. Again, statistics are not done properly, no comparison between column 1 (siControl) and column 4 (siBRG1/Chr4)

BRG1 and CHD4 are known to co-occupy numerous regulatory regions to control the same gene or pathway[6], but their mutual regulatory relationship is less explored. Recent research showed that CHD4 is required for *Brg1* expression during early bovine development[7]. As the Reviewer pointed out, our new findings indicate that knocking down BRG1 decreases *Chd4* transcripts in MS1 cells (**Fig. 5A, *p*=0.022**). We have also seen a similar trend in C166 cells—a murine extra-embryonic endothelial cell line[8] (**Fig. R1, *p*=0.0470**). These findings suggest that BRG1 may directly regulate *Chd4* transcription in mouse endothelial cells, although the specific mechanism by which it does so would require further investigation.

© 2026. Published by The Company of Biologists under the terms of the Creative Commons Attribution License (https://creativecommons.org/licenses/by/4.0/).

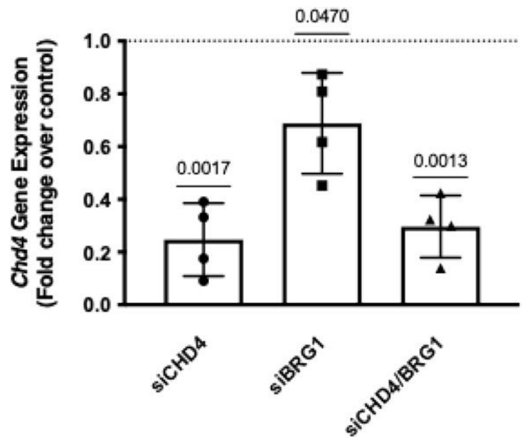

Fig. R1: *Chd4* transcripts following knockdown of CHD4 and/or BRG1 in the C166 extra-embryonic mouse EC line. qRT-PCR analysis showing *Chd4* gene transcripts in C166 cells at 24 hours after knockdown. The dotted line represents the relative expression of transcripts after treatment with a control siRNA. Data are represented as mean (±SD). Statistics: 1-sample *t* and Wilcoxon tests (comparing each knockdown against control siRNA).

Although we observed a trend toward upregulation of *Icam1* in the siBRG1/CHD4 group in Fig. 5A, statistical analysis indicated that the difference between the siControl and siBRG1/CHD4 groups was insignificant (p=0.063). We have added this information to the updated panel in **Fig. 5A**.

Reviewer 2: My comments were sufficiently addressed, and I have no further comments.

Reviewer 3: The authors have addressed many points, but some clarification is necessary.

1. Considering the IF images presented in R1 (which should be incorporated into the manuscript rather than solely as a rebuttal figure), could the developing sinusoids perhaps be marginally wider in the Chd4-KO condition? This consideration might be significant as it could influence hemodynamics within the rapidly expanding liver. A comparison with experimental measurements appears relevant to me, along with a discussion of its potential relevance to the peripheral bleeding phenotype. Does it promote blood leakage, or suggest impaired endothelial barrier stability and/or extracellular matrix/capsule integrity?

Thank you for these suggestions. We have now included the images of cellular components from our previous Fig. R1 into our new Fig. S6. To determine whether *Chd4-ECko* mutants have dilated liver sinusoids, we performed LYVE-1 whole-mount staining (which labels liver sinusoidal ECs) and quantified vessel diameters in E13.5 control and mutant livers. We found a significant increase in sinusoidal diameters in *Chd4-ECko* livers compared to controls (**Fig. R2**). Notably, sinusoidal diameters were also enlarged in *Brg1/Chd4-ECdko* livers (**Fig. R2**). Therefore, although *Chd4-ECko* livers exhibit dilated sinusoids, this dilation is not a likely cause of red blood cell accumulation and liver degeneration, since these phenotypes were rescued independently of sinusoid dilation in *Brg1/Chd4-ECdko* livers. This point has been added to the discussion (lines 396-403).

© 2026. Published by The Company of Biologists under the terms of the Creative Commons Attribution License (https://creativecommons.org/licenses/by/4.0/).

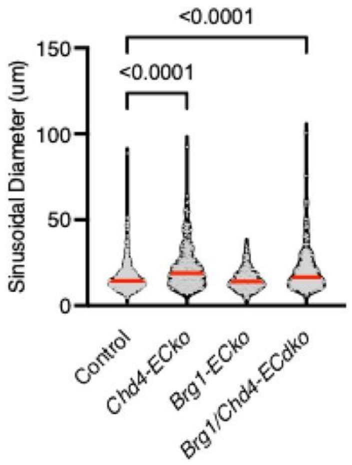

Fig. R2: Sinusoidal capillary diameters in control and mutant livers at E13.5. Violin plot of sinusoidal diameters; ≥ 138 capillaries/embryo measured. Control: n=4; mutants: n=2 biological replicates. Statistics: Kruskal-Wallis with Dunn's multiple comparisons test.

2. Since no antagonistic effect was observed in HUVECs, external datasets of human LSEC-like cells derived from iPSCs, alone or in organoids, could be assessed and for Chd4 expression in human settings, e.g. https://www.nature.com/articles/s41598-024-64195-1, or https://www.sciencedirect.com/science/article/pii/S2589555924000247 . At any rate, a revised title should reflect that the paper's observation relates to mice.

As suggested, we analyzed single-cell transcriptomic profiles from online databases and found that *CHD4* is expressed in both fetal and adult human liver ECs (**Figs. R3** and **R4**) [9,10].

© 2026. Published by The Company of Biologists under the terms of the Creative Commons Attribution License (https://creativecommons.org/licenses/by/4.0/).

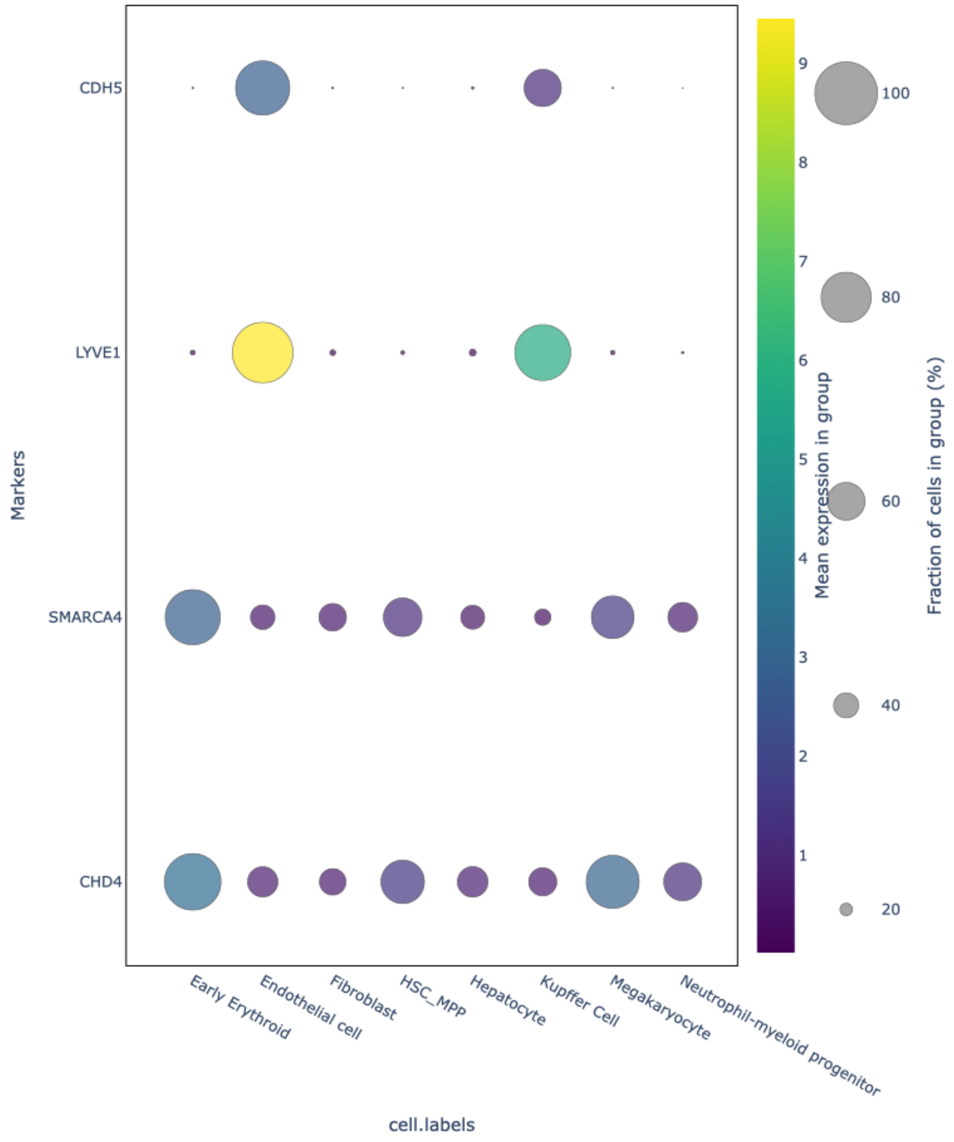

Fig. R3: *CHD4* and *BRG1* (*SMARCA4*) are expressed in human fetal liver endothelial cells. Dot plot of *CHD4*, *SMARCA4*, *LYVE1*, and *CHD5* expression in human fetal liver cells. Adapted from PMC6861135[9].

© 2026. Published by The Company of Biologists under the terms of the Creative Commons Attribution License (https://creativecommons.org/licenses/by/4.0/).

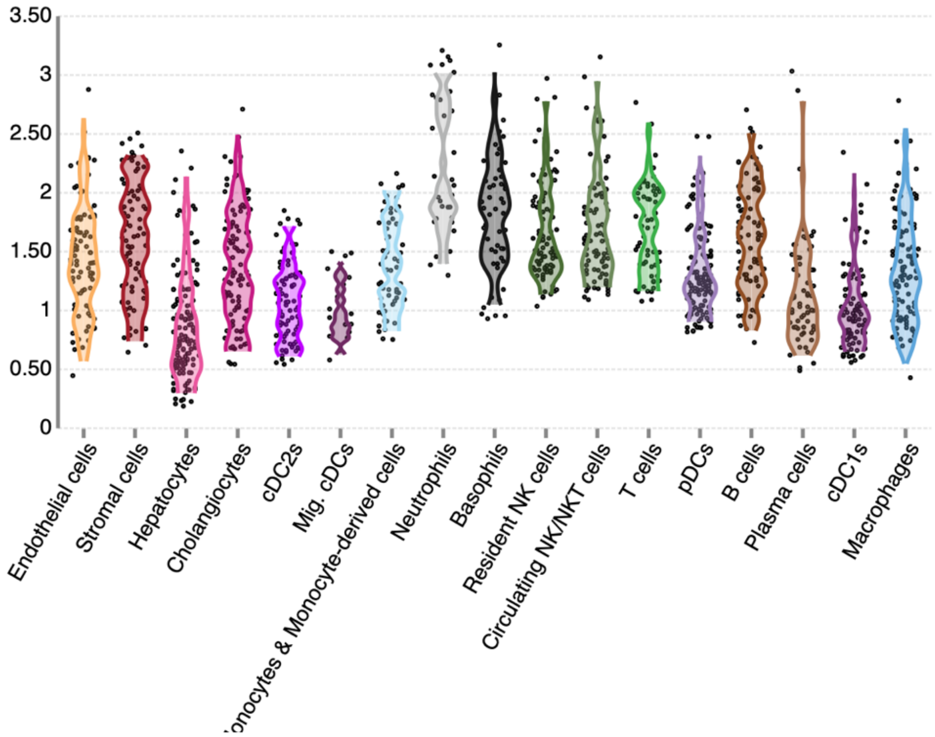

Fig. R4: *CHD4* is expressed in human liver ECs. Violin plot of *CHD4* expression in adult human liver cells. Adapted from PMC8809252[10].

Altogether, this confirmation of *CHD4* expression in human hepatic ECs does not shed further insight into why BRG1 and CHD4 failed to demonstrate antagonistic regulation of *PLAUR* and *ICAM1* transcripts in cultured HUVECs in our hands. Therefore, we have updated the title of our manuscript according to the Reviewer's suggestion: "Plasmin and sterile inflammation jointly drive fatal embryonic liver degeneration in endothelial chromatin remodeler mutant mice."

3. Minor: the discussion should be updated to note that models of the fetal liver, including a heterogeneous mixture of immune cells, albeit in their first iterations, do exist, https://pubmed.ncbi.nlm.nih.gov/41418844/
We have included this information in the "Limitations" section of the discussion (see lines 424-427).

4. Minor: A discussion should be provided regarding whether subsets of macrophages may be involved in the rescue phenotype of the double-KO-mice. This is relevant because the staining in R1 does not permit elucidation as to whether specific repair or anti-inflammatory subsets of yolk sac-derived macrophages are involved in mitigating the repair.
We appreciated this suggestion and have included this in the "Limitations" section (see lines 427-430).

In summary, we have included the following changes to this revised manuscript:

- **Relabeling of Fig. 2F:** We clarified the genotypes of *Plg*-deficient *Chd4* mutant embryos dissected from $Plg^{+/-};Chd4^{fl/fl}$ dams at E17.5.
- **Relabeling of Fig. S3B:** We clarified the genotypes of *Plg*-deficient *Chd4* mutant embryos dissected from $Plg^{-/-};Chd4^{fl/fl}$ dams at E17.5.
- **Regraphing of Fig. 5A:** We included non-significant statistical comparisons of *Icam1* transcripts in siControl- versus siBRG1/CHD4-treated MS1 ECs.
- **Relabeling of Fig. 6B:** We clarified the genotypes of *Plg*-deficient *Chd4* mutant embryos dissected at E17.5 from $Plg^{+/-};Chd4^{fl/fl}$ dams treated with saline or carprofen.
- **Relabeling of Fig. 6C-F:** We clarified the genotypes of livers sectioned and stained from *Plg*-deficient *Chd4* mutant embryos dissected at E14.5 from $Plg^{+/-};Chd4^{fl/fl}$ dams treated with

© 2026. Published by The Company of Biologists under the terms of the Creative Commons Attribution License (https://creativecommons.org/licenses/by/4.0/).

saline or carprofen.

- **New Fig. S6:** Representative images showing cellular marker expression in control and mutant livers (from our previous Fig. R1).
- **Shortened abstract:** Per *Development* requirements
- **Discussion additions:** We added new information requested by the Reviewers, including a new "Limitations of study" section.

References for this revision:

1. Crosswhite PL, Podsiadlowska JJ, Curtis CD, Gao S, Xia L, Srinivasan RS, Griffin CT. CHD4-regulated plasmin activation impacts lymphovenous hemostasis and hepatic vascular integrity. *J Clin Invest*. 2016;126:2254-2266. doi: 10.1172/JCI84652
2. Wu ML, Wheeler K, Silasi R, Lupu F, Griffin CT. Endothelial Chromatin-Remodeling Enzymes Regulate the Production of Critical ECM Components During Murine Lung Development. *Arterioscler Thromb Vasc Biol*. 2024;44:1784-1798. doi: 10.1161/ATVBAHA.124.320881
3. Wiley MM, Muthukumar V, Griffin TM, Griffin CT. SWI/SNF Chromatin-Remodeling Enzymes Brahma-Related Gene 1 (BRG1) and Brahma (BRM) Are Dispensable in Multiple Models of Postnatal Angiogenesis But Are Required for Vascular Integrity in Infant Mice. *J Am Heart Assoc*. 2015;4. doi: 10.1161/JAHA.115.001972
4. Satoh S, Nussler AK, Liu ZZ, Thomson AW. Proinflammatory cytokines and endotoxin stimulate ICAM-1 gene expression and secretion by normal human hepatocytes. *Immunology*. 1994;82:571-576.
5. Sano K, Nagaki M, Sugiyama A, Hatakeyama H, Ohnishi H, Muto Y, Moriwaki H. Effects of cytokines on the binding of leukocytes to cultured rat hepatocytes and on the expression of ICAM-1 by hepatocytes. *Dig Dis Sci*. 1999;44:796-805. doi: 10.1023/a:1026682329876
6. Morris SA, Baek S, Sung MH, John S, Wiench M, Johnson TA, Schiltz RL, Hager GL. Overlapping chromatin-remodeling systems collaborate genome wide at dynamic chromatin transitions. *Nat Struct Mol Biol*. 2014;21:73-81. doi: 10.1038/nsmb.2718
7. Li S, Xiao L, Shi Y, Hu B, Wu X, Wang S, Zhang K. CHD4 Ensures Bovine Early Embryogenesis by Restricting Developmental Gene Expression and Maintaining BRG1 Expression. *FASEB J*. 2026;40:e71522. doi: 10.1096/fj.202503458R
8. Wang SJ, Greer P, Auerbach R. Isolation and propagation of yolk-sac-derived endothelial cells from a hypervascular transgenic mouse expressing a gain-of-function fps/fes proto-oncogene. *In Vitro Cell Dev Biol Anim*. 1996;32:292-299. doi: 10.1007/BF02723062
9. Popescu DM, Botting RA, Stephenson E, Green K, Webb S, Jardine L, Calderbank EF, Polanski K, Goh I, Efremova M, et al. Decoding human fetal liver haematopoiesis. *Nature*. 2019;574:365-371. doi: 10.1038/s41586-019-1652-y
10. Guilliams M, Bonnardel J, Haest B, Vanderborght B, Wagner C, Remmerie A, Bujko A, Martens L, Thone T, Browaeys R, et al. Spatial proteogenomics reveals distinct and evolutionarily conserved hepatic macrophage niches. *Cell*. 2022;185:379-396 e338. doi: 10.1016/j.cell.2021.12.018

Second decision letter

MS ID#: dev.205538R1

MS Title: Plasmin and sterile inflammation jointly drive fatal embryonic liver degeneration in endothelial chromatin remodeler mutant mice

Authors: Meng-Ling Wu; Yelyzaveta Rudenko; Courtney Griffin

Dear Dr Griffin,

I am happy to tell you that your manuscript has been accepted for publication in Development, pending our standard publication integrity checks.

© 2026. Published by The Company of Biologists under the terms of the Creative Commons Attribution License (https://creativecommons.org/licenses/by/4.0/).
